# Subunits of an E3 Ligase Complex as Degrons for Efficient Degradation of Cytosolic, Nuclear, and Membrane Proteins

**DOI:** 10.1021/acssynbio.3c00588

**Published:** 2024-02-26

**Authors:** Anže Verbič, Tina Lebar, Arne Praznik, Roman Jerala

**Affiliations:** Department of Synthetic Biology and Immunology, National Institute of Chemistry, Ljubljana 1000, Slovenia

**Keywords:** synthetic biology, degrons, control of protein expression, E3 ligase

## Abstract

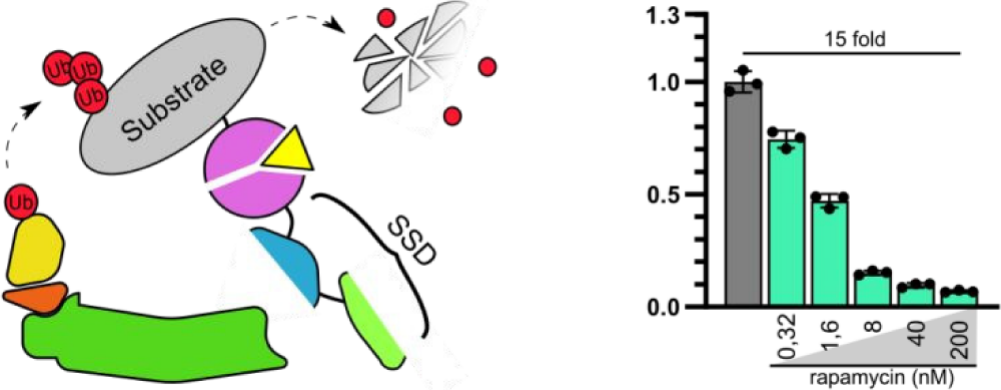

Protein degradation is a highly regulated cellular process crucial to enable the high dynamic range of the response to external and internal stimuli and to balance protein biosynthesis to maintain cell homeostasis. Within mammalian cells, hundreds of E3 ubiquitin ligases target specific protein substrates and could be repurposed for synthetic biology. Here, we present a systematic analysis of the four protein subunits of the multiprotein E3 ligase complex as scaffolds for the designed degrons. While all of them were functional, the fusion of a fragment of Skp1 with the target protein enabled the most effective degradation. Combination with heterodimerizing peptides, protease substrate sites, and chemically inducible dimerizers enabled the regulation of protein degradation. While the investigated subunits of E3 ligases showed variable degradation efficiency of the membrane and cytosolic and nuclear proteins, the bipartite SSD (SOCSbox-Skp1(ΔC111)) degron enabled fast degradation of protein targets in all tested cellular compartments, including the nucleus and plasma membrane, in different cell lines and could be chemically regulated. These subunits could be employed for research as well as for diverse applications, as demonstrated in the regulation of Cas9 and chimeric antigen receptor proteins.

## Introduction

Protein degradation is an essential cellular function, regulating many cellular processes. It plays a critical role in removing damaged and misfolded proteins, enables response on the abundance of transcription factors, facilitates cell cycle progression, and, in general, ensures the timely and coordinated disposal of proteins that are no longer needed, thus ensuring a dynamic response to the changing conditions and maintaining cellular homeostasis.

**Figure 1:**
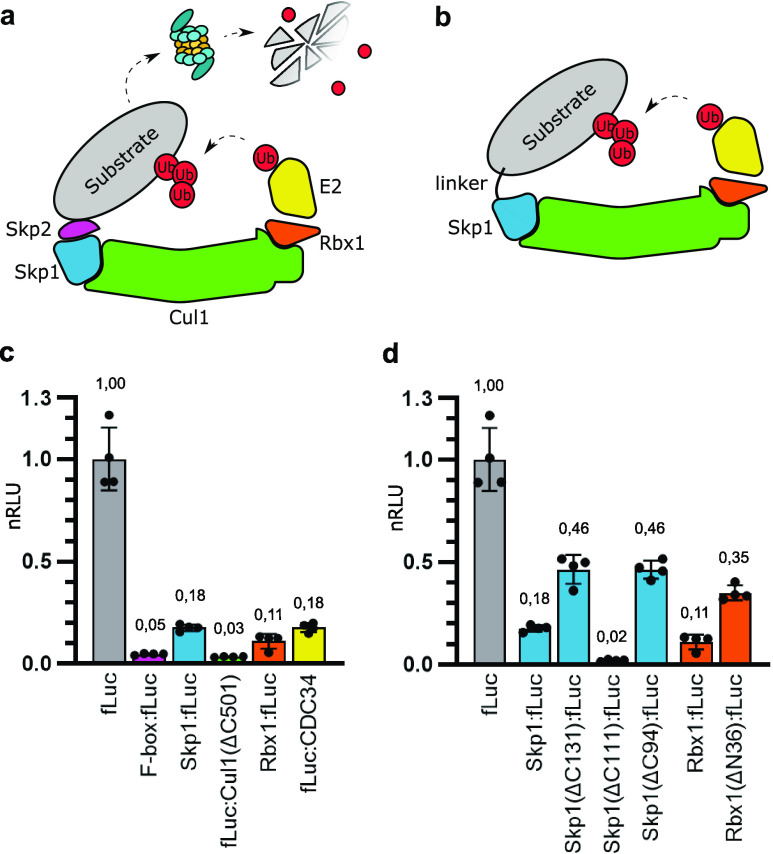
Design of degrons based on the four subunits of the SCF-Skp2 E3 ligase complex. (a) Schematic representation of an SCF-Skp2 E3 ligase complex. The complex consists of a central Cul1 scaffold protein that binds Rbx1 on its C terminus, which enables the binding of E2-Ub intermediates. At the N-terminus, Cul1 binds Skp1, an adapter protein that enables the binding of different F-box proteins that bind different protein substrates. (b) Fragments of SCF E3 ligase as degrons. Genetic fusion of a substrate with E3 ligase-derived genes leads to ubiquitination and subsequent degradation of the substrate protein. (c) Plasmids expressing the substrate firefly luciferase with and without genetic fusion to subunits of SCF-Skp2 E3 ligase were transfected into HEK293T cells, and relative luciferase activity was measured 48 h post-transfection. (d) Plasmids expressing genetic fusions of firefly luciferase and deletion mutants of Skp1 and Rbx1 with removed domains with which they interact with Skp2 and Cul1, respectively, were transfected into HEK293T cells, and relative luciferase activity was measured 48 h post transfection. Values in (c) and (d) panels represent the mean ± SD of four cell culture experiments and are normalized to the expression of luciferase without degron fusions. Transfection plasmid mixtures are listed in Table S1. nRLU means normalized relative luciferase units.

The majority of proteins in mammalian cells are degraded through the ubiquitin-proteasome pathway. The degradation of proteins into short peptides is catalyzed by a 26S proteasome, which recognizes and degrades cellular proteins tagged with a polyubiquitin chain.^1^ The polyubiquitin chain is covalently attached to a surface-exposed lysine residue of a polypeptide substrate by an enzyme cascade consisting of E1, E2, and E3 proteins. Briefly, E1 activates the ubiquitin (Ub) in an ATP-dependent reaction by forming a thioester with the Ub C terminus and then transfers the Ub to a cysteine at the E2 to form an E2-Ub intermediate. E3 ligase binds the E2-Ub intermediate and a polypeptide substrate and catalyzes the formation of an iso-peptide bond between the Ub and a lysine residue of the polypeptide substrate and extends the chain of conjugated Ub substrates, which constitutes the proteasomal degradation signal^2,3^ (Figure 1a). E3 ligases are key regulators of the Ub proteasome pathway, controlling the specificity and efficiency of ubiquitin transfer and consequently degradation of Ub-tagged proteins.^2,3^ The existence of more than 600 ubiquitin ligases demonstrates that this is a highly regulated process with high specificity and targeted regulation of different substrates.

Recently, methods have been developed to control the degradation of selected proteins to study their function,^4−6^ design synthetic circuits,^7,8^ regulate gene expression,^9,10^ and degrade disease-causing and other nondesired proteins.^11,12^ To facilitate degradation, different destabilizing domains (or “degrons”) have been developed that guide selected proteins to the degradation machinery. These rely on the interaction with cellular E3 ligases,^13−15^ 26S proteasome,^7,16^ chaperones,^17−19^ or through currently unknown mechanisms.

Here, we systematically investigated the protein subunits of the SCF-Skp2 E3 ligase complex as tags for the degradation of selected proteins. We demonstrate that their degradation can be further modulated by the introduction of modular coiled-coil domains and small molecules. We construct a composite degron that facilitates efficient degradation of proteins in the cytosol and plasma membrane as well as in nuclei in different cell lines.

## Results

### Design of Degrons Based on the SCF-Skp2 E3 Ligase Complex

The vast majority of human E3 ligases are RING-type (Really Interesting New Gene) E3 ligases. RING-type E3s are characterized by the RING domain, which enables direct transfer of Ub from E2 to the substrate protein, without an E3-Ub intermediary.^20^ SCF-Skp2 E3 ligase is a structurally well-characterized mammalian RING-type E3 ligase. This modular complex comprises at its center a long curved Cul1 scaffold protein, which binds Rbx1 and Skp1 at the C- and N terminus, respectively (Figure 1a). Rbx1 contains a RING domain that enables the recruitment of an E2-Ub intermediate to the Cul1-Rbx1 catalytic core complex. Skp1 acts as an adapter protein, enabling the binding of different F-box proteins, such as Skp2. F-box adapter proteins contain a highly conserved N terminal F-box motif that binds to Skp1, while the C terminus is variable and is responsible for binding different specific protein substrates. In this highly modular assembly, the F-box proteins control the substrate specificity, Rbx1 enables efficient E2-Ub exchange, and Cul1 positions the substrate and E2-Ub in an optimal spatial arrangement for Ub transfer.^20−22^ This assembly is also highly dynamic and enables the exchange of both domains introducing Ub moieties and substrate-binding F-box proteins, which enables high processivity.

As the key factor for the transfer of Ub and formation of the polyubiquitin chain on the substrate is the proximity of the substrate to the E2-Ub, we hypothesized that the fusion of subunits of SCF-Skp2 ligase to a substrate would lead to its polyubiquitination and its subsequent degradation (Figure 1b). To explore this hypothesis, we genetically fused four domains of the SCF-Skp2 E3 ligase complex (Skp2 F-box, Skp1, Rbx1, and C terminus of Cul1) to a firefly luciferase reporter protein. Furthermore, we genetically fused CDC34 E2 to firefly luciferase to explore if fusing E2 directly to a substrate could enable its degradation. We chose CDC34 as it is known to associate with SCF family E3 ligases and was shown to catalyze the generation of Lys48-linked polyubiquitin chains, which enable recruitment of the substrate to the mammalian 26S proteasome.^23,24^ As shown in Figure 1c, genetic fusions of all SCF subunits and CDC34 with firefly luciferase resulted in a significant decrease in the luciferase activity, suggesting augmented degradation of the luciferase, with F-box and Cul1(ΔC501) exhibiting the highest decrease in luciferase activity (plasmid transfection mixtures are described in Table S1 and amino acid sequence of transfected constructs is in Table S2). As these two domains were modified by only retaining Skp1-interacting and Rbx1-interacting domains of Skp2 and Cul1, respectively, we wanted to see if we could similarly improve the degradation mediated by Skp1. Skp1 binds Skp2 through four C-terminal helices, so we prepared three variants of Skp1 with two (Skp1(ΔC131)), three (Skp1(ΔC111)), and all four (Skp1(ΔC94)) helices removed (Note S1). The Skp1 variant with a deletion of three helices showed a significant improvement in degradation over the full-length Skp1, while the other two variants exhibited reduced degradation (Figure 1d). Similarly, we prepared an Rbx1 variant with a deletion of an N-terminal beta-sheet, removing most of its contacts with Cul1; however, it showed reduced degradation compared to a native Rbx1 (Figure 1d, Note S1). The addition of a proteasome inhibitor reduced the degradation potency of all of the tested degrons, indicating their dependency on the UPS degradation pathway (Figure S6a–c).

These results indicate that the subunits of the SCF-Skp2 E3 ligase can act as potent degrons to destabilize substrates. Surprisingly, all elements from the F-box substrate receptor, Skp1 adapter, Cul1 scaffold, and Rbx1 adapter to the CDC34 E2 protein tested here exhibited high degradation of the reporter substrate. By removing substrate receptor-binding motifs from Skp1, we managed to further improve its degradation potency.

### Control of SCF-Skp2 E3 Ligase-Derived Degrons

**Figure 2:**
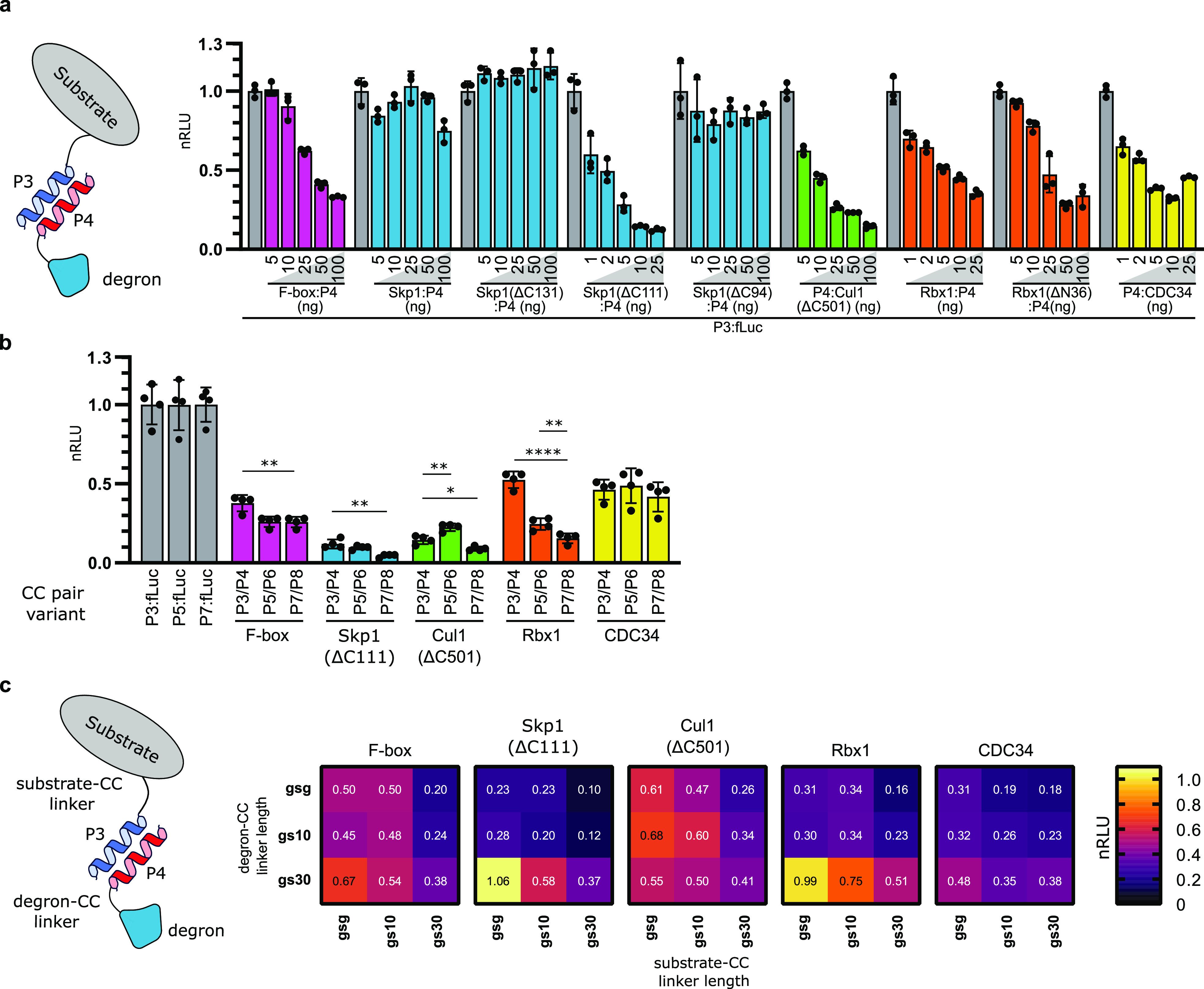
Control of SCF-Skp2 E3 ligase-derived degrons via coiled-coil-fusion and linker length.(a) Coiled-coil (CC)-mediated degradation. The luciferase substrate with P3 and degron with P4 CC fusion proteins were cotransfected into HEKT293T cells at an increasing ratio of degron to substrate plasmid construct. (b) Effect of CC affinity on the degradation rate. Best performing degrons from panel (a) were tested with different CC heterodimers, P3/P4, P5/P6, and P7/P8, with decreasing affinities. (c) Effect of linker length between the CC dimer and both the substrate and degron. Luciferase substrates with flexible GS linkers 3, 10, and 30 amino acids in length between the substrate and coiled coil were cotransfected with degrons with the same linkers between coil and degron segments. Heat map data are presented in Figure S9. Plasmids expressing the described proteins were cotransfected into HEK293T cells. Luciferase activity was measured 48 h post-transfection. Values represent the mean ± SD of three cell culture experiments and are normalized to the expression of luciferase without degron fusions. Transfection plasmid mixtures are listed in Table S1. Statistical analysis was conducted by using a two-sided unpaired *t* test. nRLU means normalized relative luciferase units.

Next, we sought a way to control protein degradation mediated by SCF-Skp2 E3 ligase-derived degrons. The ability to modulate the degradation rate could enable control of the protein half-life and make it applicable to a wider range of uses. We first decided to modulate degradation by decoupling the target substrate and degrons and expressing them separately, to enable control of the substrate degradation by modulating the expression of the degron, and to prevent degradation of the degron domains identified above as direct fusion. This strategy could also enable the degradation of multiple target proteins to engage the same type of degron. To ensure that the substrate is connected to degrons, we used designed coiled-coil (CC) heterodimers. CC heterodimers have been previously used to build complex modular 3D structures^25,26^ and logic circuits^27,28^ and to regulate cellular processes.^29−31^ They can be designed to be highly orthogonal to other CC pairs and natural proteins and have a tunable affinity between partner peptides.^31^ We prepared genetic fusions of all degrons with a P4 CC-forming peptide and luciferase substrate with a complementary P3 peptide^32^ (Figure 2a). By increasing the amount of cotransfected CC-degron fusion protein, we were able to modulate substrate degradation with most degrons (Figure 2a). With F-box, Skp1(ΔC111), Cul1, Rbx1, Rbx1(ΔN36), and CDC34, a clear CC-degron concentration-dependent degradation can be observed, but not for Skp1, Skp1(ΔC94), and Skp1(ΔC131) domains in this setting. To confirm that the substrate was indeed degraded, we analyzed the amount of the substrate protein in cell lysates after cotransfection with different degrons using Western blot, which showed that the amount of protein correlated with the luciferase activity (Figure S7). The addition of proteasome inhibitor reduced substrate degradation for all tested degrons (Figure S8). Modulation of expression of CC-degron fusion proteins can therefore be used to control the degradation of the target substrate by employing CC-mediated interactions between the degron and substrate.

One useful aspect of using CC heterodimers in this setting is, as mentioned, that they can be designed to have a desired affinity. The affinity between the substrate and E3 ligase is an important factor in designing synthetic bifunctional degraders (PROTACs).^33^ The use of CC heterodimers enabled us to change the affinity by selecting the appropriate coiled-coil heterodimer. The exchange of the substrate from the E3 ligase can increase the processivity and provide faster turnover. To explore if we could control degradation by changing CC heterodimers, we tested three different CC heterodimers (P3/P4, P5/P6, P7/P8) with decreasing affinities.^29,32^ We observed that by using CC heterodimers with lower affinity, the substrate degradation in general increased (Figure 2b). With Skp1(ΔC111) and Rbx1, we saw the largest increase in degradation by using CC heterodimers with lower affinity with a 2,4-fold and 3,4-fold increase, respectively. This increase in degradation rate was still significant but less pronounced with the F-box.

Optimization of linkers connecting different proteins or protein domains in fusion proteins is frequently required for modular designs in synthetic biology. Linker lengths can range from just a few to dozens of amino acids, and optimal length can depend on the type of protein and the specific application of the fusion protein. Since the efficient ubiquitin transfer might require some rigidity of the substrate–E3 ligase complex, we decided to explore the effect of linker lengths between coiled coils and both the substrate and degron.^22^ We tested three different flexible glycine-serine (gs) linkers: 3, 10, and 30 amino acid residues (Figure 2c). The effect of linker length between both the substrate and degron to the CC heterodimer seemed to depend on the degron, with some degrons exhibiting a higher sensitivity of linker length on degradation than others. In general, the longer linker between the substrate and CC heterodimer lead to higher degradation, while the shorter linker length between the degron and CC heterodimer resulted in more efficient degradation.

### Development of Inducible Degradation Systems

**Figure 3:**
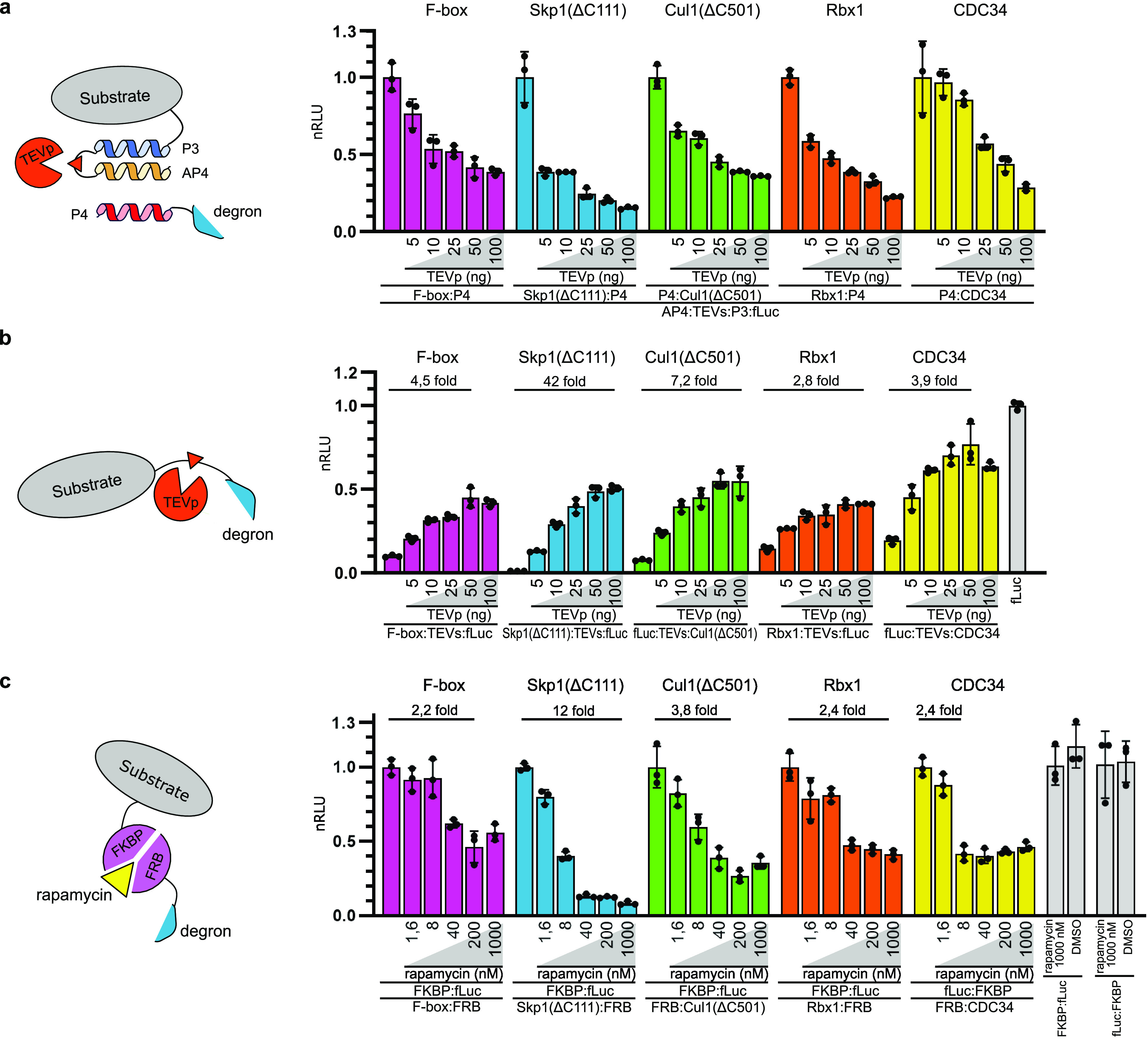
Development of diverse inducible degradation systems.(a) Design of a system of activation of degradation using viral proteases. Luciferase substrates are genetically fused with a P3 coil to an inhibitory AP4 coil through a linker containing a TEV protease cleavage site. Proteolytic cleavage of the linker enables the dissociation of the AP4 coil and the association of a P4 displacing coil, which is genetically fused with a degron, enabling the degradation of the substrate. Substrate and degron plasmid constructs were cotransfected with increasing amounts of the protease construct. (b) Design of a system of inhibition of degradation using viral proteases. Luciferase substrates are genetically fused with the degron through a linker containing a TEV protease cleavage site. With linker cleavage, the degron dissociates from the substrate, inhibiting substrate degradation. Substrate and degron plasmid constructs were cotransfected with increasing amounts of protease construct. (c) Control of substrate degradation using chemically induced dimerization. The luciferase substrate was genetically fused with the FKBP domain and degrons with the FRB domain, enabling dimerization of the substrate and degron in the presence of rapamycin. Plasmids expressing the described proteins were cotransfected into HEK293T cells. Rapamycin or DMSO were added to cell culture 24 h post-transfection (c), and luciferase activity was measured 48 h post-transfection. Rapamycin or DMSO did not have an effect on substrate expression without coexpression of the degron construct (Figure S10a). Values represent the mean ± SD of three cell culture experiments and are normalized to (a) expression of luciferase without transfected TEV protease, (b) expression of luciferase without degron fusion, and (c) expression of luciferase and degron fusion proteins without added rapamycin. Transfection plasmid mixtures are listed in Table S1. nRLU means normalized relative luciferase units.

Next, we set out to develop a system of inducible degradation of target proteins using degrons derived from the SCF-Skp2 E3 ligase. Proteolysis using highly specific viral proteases has been used for the construction of synthetic circuits to control protein expression,^34−36^ signal sensing,^37−39^ protein translocation,^28,40^ and construction of signaling networks.^27,36^ Due to the designable nature of coiled coils, a device can be constructed where an autoinhibitory intramolecular coil inhibits the binding of a competing coil to the primary coil. The proteolytic cleavage of an autoinhibitory coil from the primary coil enables the binding of the competing coil to the primary coil.^27^ We used this strategy to design a device for inducible degradation by genetic fusion of a degron with the competing coil and the primary coil with autoinhibitory coil fused to the substrate, where the primary and inhibitory coil were connected through a peptide linker containing the substrate sequence of a highly specific TEV protease (Figure 3a). Linker cleavage facilitates the dissociation of the inhibitory coil and binding of a CC-degron fusion protein to the substrate and activating its degradation. The cleavage of an inhibitory coil indeed enabled efficient regulation of the degradation of the substrate protein with all of the tested CC-degron fusion proteins in a protease-concentration-dependent manner (Figure 3a).

Similarly, to achieve a protease-inducible inhibition of degradation, we designed a device where the degron is fused directly to the substrate through a linker that contains a protease cleavage site. Linker cleavage in this device leads to a disassociation of the degron from the substrate protein, effectively inhibiting its degradation (Figure 3b). Using this approach, we could control the degradation of the substrate using all SCF-Skp2 E3 ligase-derived degrons, while the dynamic range was greatest with Skp1(ΔC111).

Next, we wanted to implement chemically induced dimerization (CID) to regulate degradation using small molecule inducers. CID is used to control protein dimerization by reconstitution of protein domains upon the binding of small molecules. The use of small molecules is attractive as it enables temporal control of biological processes and can be used both in vitro and in vivo^41,42^ and has been implemented in several designed degradation systems.^18,43−47^ To achieve the control of protein degradation through CID, we employed a well-established system of rapamycin-inducible reconstitution of FKBP and FRB protein domains^48^ (Figure 3c). Expressing the substrate in genetic fusion with FKBP and SCF-Skp2-derived degrons with FRB and inducing dimerization with rapamycin, we observed successful degradation of the substrate with all degrons with the highest efficiency by using Skp1(ΔC111), where we observed a 12-fold reduction in luciferase activity.

These described diverse modes of regulation allow the incorporation of protein degradation through selected degron domains for implementation into various cellular circuits.

### Inducible Degradation of Target Proteins Localized to the Plasma Membrane or Nucleus

**Figure 4:**
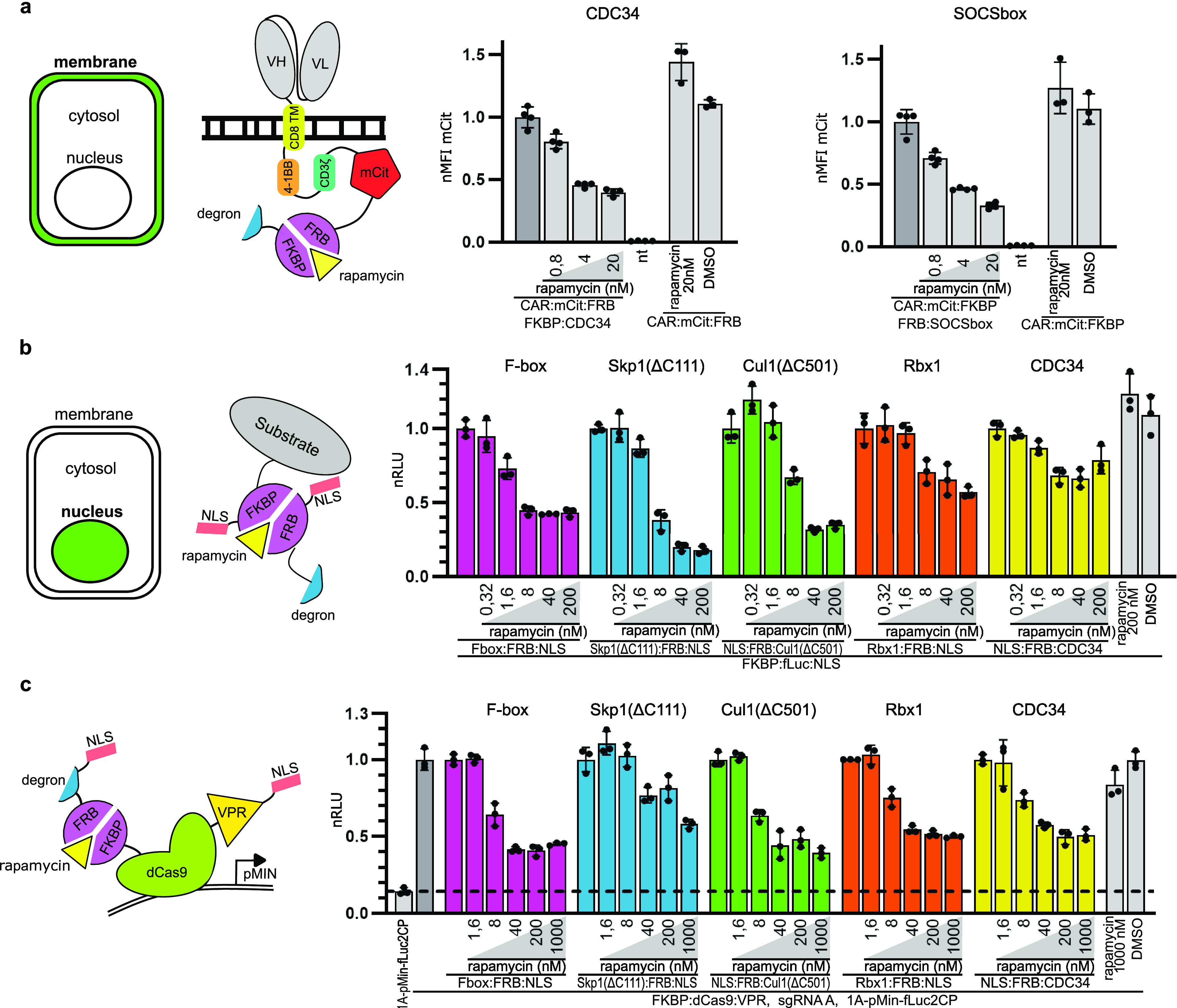
Inducible degradation of target proteins localized to the plasma membrane and the nucleus. (a, left) Design of a membrane degradation reporter protein. Second generation anti-CD19 CAR was genetically modified to include a C-terminal mCitrine fluorescent protein to monitor degradation and an FRB domain, which enabled us to induce degradation of CARs using rapamycin. (a, right) Degradation of CAR protein by CDC34 and SOCSbox degrons. The CAR substrate and degron plasmids were expressed in HEK293T cells, and cells were treated with different concentrations of rapamycin and analyzed by flow cytometry. (b) Degradation of the luciferase substrate in the nucleus. The luciferase substrate and degrons were genetically fused with a nuclear localization sequence (NLS) enabling the translocation of both elements into the nucleus. (c) Degradation of the dCas9 nuclear protein. Left: The dCas9:VPR:NLS transcriptional activator was genetically fused with the FKBP domain, while the NLS tag was added to degrons, enabling interaction with the two proteins inside the nucleus. Right: dCas9:VPR:NLS, sgRNA, and degron plasmids were cotransfected together with the target plasmid with the fLuc reporter gene under the control of a *Pmin* promoter. The dashed line represents the value of luciferase expression in the absence of the dCas9:VPR transactivator. Plasmids expressing the described proteins were cotransfected into HEK293T cells. Rapamycin was added to cell culture 24 h post-transfection, and luciferase activity (b, c) or fluorescence (a) was measured 48 h post-transfection. Values represent the mean ± SD of three (b, c) or four (a) cell cultures experiments and are normalized (b) to the expression of luciferase and degron fusion proteins without added rapamycin (c) to the expression of target plasmid with fLuc reporter gene under the control of a *Pmin* promoter, sgRNA, dCas9:VPR:NLS, and degron construct without rapamycin. Transfection plasmid mixtures are listed in Table S1. nRLU means normalized relative luciferase units; nMFI means normalized mean fluorescence intensity; and nt means nontransfected cells.

The above-described results demonstrated the degradation of cytosolic substrates using SCF-Skp2 E3 ligase-derived degrons. Next, we wanted to explore if these degrons can degrade membrane and nuclear proteins, which play distinct roles in the cell. As a model for the degradation of membrane proteins, we chose chimeric antigen receptors (CARs). Controlling the abundance of CAR proteins at the surface of engineered T cells could be used to increase the safety of CAR-based immunotherapy by inhibiting the side effects of excessive activation such as cytokine storms and neurotoxicity.^49^ We genetically fused second generation anti-CD19 CAR constructs with mCitrine fluorescent protein to monitor degradation and an FRB domain, which enabled the inducible degradation of CARs using rapamycin (Figure 4a). This CAR fusion protein was localized at the plasma membrane in HEK293T cells (Figure S11). We observed successful degradation of CARs using CDC34, while using other degrons, the degradation was generally poor (Figure 4a). To test whether this low degradation was due to the choice of E3 ligase, we explored another E3 ligase, the CRL5-SOCS2 E3 ligase complex, which efficiently degrades the growth hormone receptor (GHR), a membrane protein. In this complex, the SOCS2 protein functions as a substrate receptor protein, binding GHR in its central SH2 domain and ElonginC with its highly conserved C-terminal SOCSbox domain.^50,51^ As the F-box from the SCF-Skp2 E3 ligase complex enabled efficient degradation and the SOCSbox is functionally analogous to the F-box, we decided to explore if it could enable the degradation of CAR proteins (degron design in Note S1, Figure S5). By using SOCSbox-based constructs, we observed efficient rapamycin-dependent degradation of integral membrane CAR proteins, with similar efficiency to CDC34 degron (Figure 4a).

To explore if SCF-Skp2 E3 ligase-derived degrons would be able to degrade nuclear proteins, we genetically fused the substrate luciferase and degron constructs to a nuclear localization signal (NLS), which enables efficient transport of proteins to the nucleus.^52^ All of the tested degron constructs were able to degrade this substrate in a rapamycin concentration-dependent fashion (Figure 4b). To further demonstrate the ability of designed degrons to deplete nuclear proteins, we tested degradation of the dCas9 protein. dCas9 is a catalytically inactive mutant of Cas9, an RNA-guided endonuclease enzyme, which can be used to regulate gene expression.^53,54^ Here, we genetically fused dCas9 with a transcriptional activator domain (VPR), an nuclear localization sequence (NLS), and an FKBP domain (Figure 4c). All of the SCF-Skp2 E3 ligase-derived degrons were able to inhibit transcriptional activation mediated by dCas9-VPR in a rapamycin concentration-dependent manner, although the overall repression seemed to be lower than with the firefly luciferase substrate. Rapamycin or DMSO did not have an effect on expression of both nuclear reporter proteins without coexpression of the degron construct (Figure S10b,c).

Taken together, these results show that the choice of E3 ligase as the source of designed degron domains is important for efficient degradation of targeted proteins, localized in different cellular compartments. Degrons designed from SCF-Skp2 E3 ligase were able to efficiently degrade proteins compartmentalized in the nucleus as well as in the cytosol. Degradation of a membrane-localized CAR was less efficient with degrons derived from the SCF-Skp2 E3 ligase, with only CDC34 showing significant degradation of the CAR proteins. Utilization of an E3 ligase that was previously shown to degrade a membrane protein GHR, as the source of a degron domain, seemed to be advantageous in developing a degron for degradation of membrane proteins. Ubiquitinated membrane proteins may also be directed to the endosomal pathway and degraded there; however, the selection of the degradation pathway is not relevant in this context as long as the degradation is efficient.^55,56^

### Concatenation of Degrons Enables Rapid Degradation of Proteins in Several Cellular Compartments

**Figure 5:**
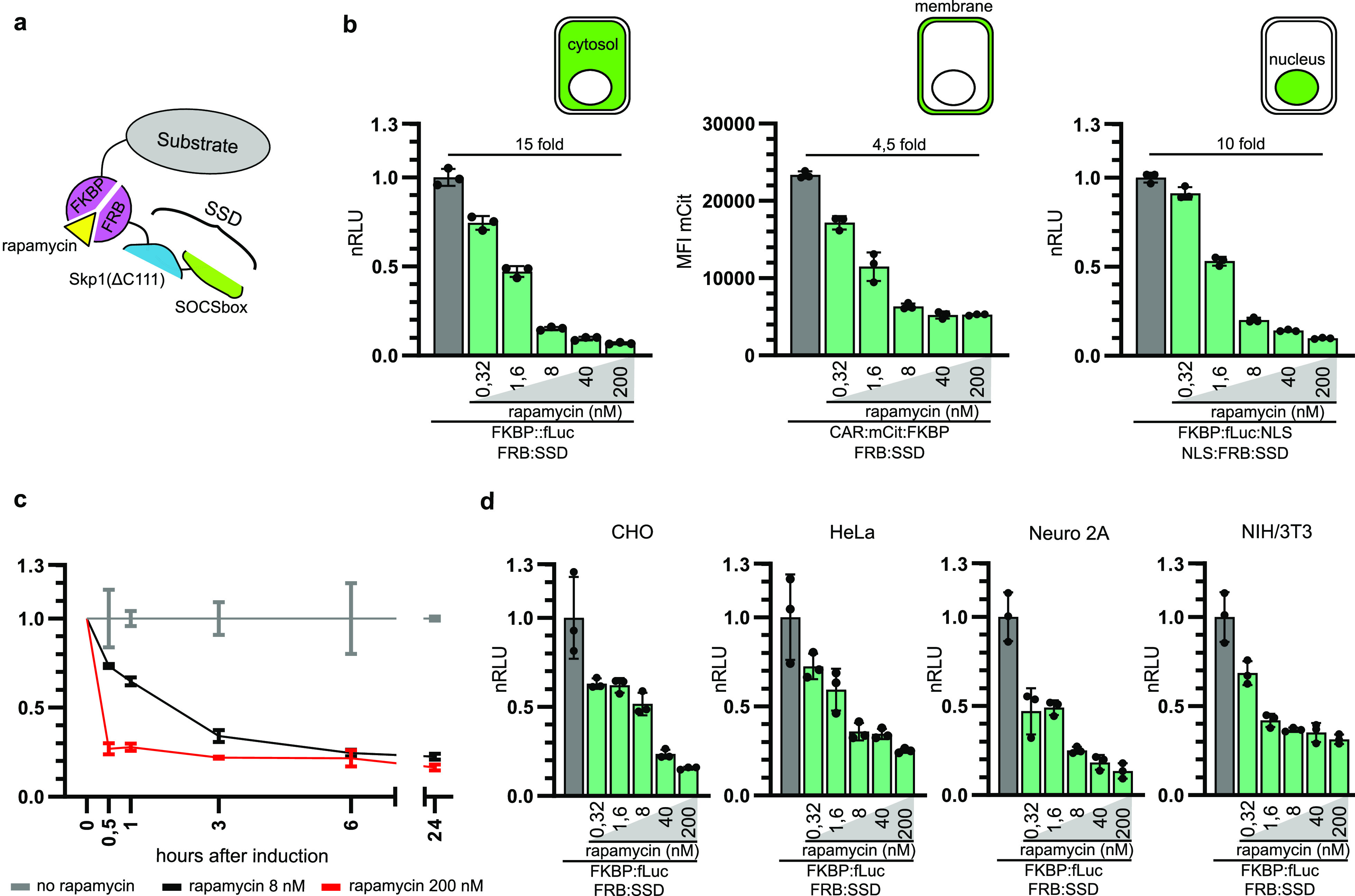
Concatenation of degrons enables rapid degradation of proteins in several cellular compartments. (a) Design of a concatenated degron by genetically fusing Skp1(ΔC111) and SOCSbox degrons with an FRB domain, termed the SSD degron. (b) Degradation of cytosolic (left), membrane (middle), and nuclear (right) proteins using the SSD degron. We used luciferase protein as the substrate for the degradation of cytosolic proteins, CAR protein for the degradation of membrane proteins, and luciferase with NLS tag for the degradation of nuclear proteins. (c) Kinetics of degradation of the cytosolic luciferase substrate using the SSD degron. Rapamycin was added to cell culture 24 h post-transfection and luciferase activity was measured at time points indicated in the figure. (d) The concatenated SSD degron enables degradation in different mammalian cell lines. Different cell lines were transfected with a cytosolic luciferase reporter protein and SSD degron, and degradation was induced with rapamycin. Plasmids expressing the described proteins were cotransfected into (b, c) HEK293T and (d) CHO, HeLa, and Neuro 2A, NIH/3T3 cells. Rapamycin was added to cell culture 24 h post-transfection, and luciferase activity (b–d) or fluorescence (b, middle) was measured 48 h post-transfection. Values represent the mean ± SD of three cell culture experiments and are normalized to the expression of luciferase and degron fusion proteins without added rapamycin in (c) of each time point. Transfection plasmid mixtures are listed in Table S1. nRLU means normalized relative luciferase units, and MFI means mean fluorescence intensity.

As presented in Figure 4, the degrons developed in this study had compartment-specific activity. This could limit their utility when used in synthetic devices, and a compartment-specific degron would need to be applied. To address this limitation, we sought to develop a degron that could target proteins for degradation in the nucleus and cytoplasm and at the plasma membrane by concatenating SOCSbox and Skp1(ΔC111) degrons into a single polypeptide chain, termed the SSD degron (Figure 5a). CDC34 was able to degrade reporter proteins in those three compartments, but due to its larger size, lower degradation efficiency, and potential toxicity, concatenation of substrate receptor proteins might be a better alternative. The concatenated SSD degron fused to an FRB domain was efficient in degrading CARs, a cytosolic protein, and a protein with a nuclear localization (Figure 5b). It enabled the efficient degradation of proteins in all three compartments in a rapamycin-dependent manner. Furthermore, the degradation of the membrane and nuclear reporter substrates was more potent than with any of the previously tested single degron constructs.

Next, we determined the kinetics of degradation by this concatenated degron. In general, the ubiquitin-proteasome system (UPS) can react fairly quickly to the changing environment and can degrade targeted proteins in a matter of minutes.^57−60^ Ideally, a system of inducible degradation of proteins can take advantage of this property of UPS and allow similarly fast degradation and thus enable rapid temporal control of protein stability. We observed that the substrate degradation using concatenated SSD degron was very fast at higher concentrations of rapamycin, achieving near-plateau levels after only 30 min (Figure 5c). A lower concentration of rapamycin slowed the degradation, where the plateau was achieved after 6 h.

E3 ligases are highly conserved due to their essential role in numerous cellular processes.^21^ We therefore expected that the concatenated degron could function in different mammalian cell lines. Indeed the degradation of cytosolic reporter substrate was comparable to degradation in the HEK293T cell line in four additional cell lines: Chinese hamster ovary, Hela, Neuro 2A, and NIH/3T3 cell lines (Figure 5d).

The concatenated SSD degron presented here represents an efficient tool for the regulation of protein stability with fast kinetics, can be used to degrade proteins in different cellular compartments and different cell lines, and could be chemically regulated, which could be used for research as well as for different applications.

## Discussion

Protein degradation through the ubiquitin-proteasome system can be a powerful tool to control protein stability to regulate cellular processes. To this end, versatile approaches to engage target proteins with the endogenous degradation machinery are needed to expand the toolbox of protein stability control, ideally for different cellular localizations. Our systematic investigation showed that all of the subunits of the SCF-Skp2 E3 ligase can be employed to facilitate the degradation of the target protein and that for some of the E3 ligase domains, their performance can be improved by taking into consideration how they interact with other proteins. In general, the degradation of our reporter substrate with genetically fused degrons was quite efficient in this setting compared to several other designed degrons.^7,61,62^ The same approach to degradation domain selection demonstrated here could be employed with other E3 ligases. We can expect that degrons engineered from their corresponding E3 ligases would retain their specific activity (tissue specificity, regulation, subcellular activity, cell cycle regulation, etc.), enabling tight regulation of degradation. Furthermore, we propose that proteolysis targeting chimeras (PROTACs) could be designed to bind the same interfaces demonstrated here, avoiding the endogenous regulation of adapter or substrate receptor proteins and potentially increasing the efficiency of their function.

The attachment of a polyubiquitin chain to the protein substrate is a complex process, and many parameters impacting this process such as the affinity of the substrate to the degron and position of the substrate lysine residues have already been described.^60,63−65^ Here, we investigated the factors that impact the performance of SCF-Skp2 E3 ligase-derived degrons interacting with the substrate through coiled-coil domains. The designable nature of coiled-coil domains enabled us to modulate the affinity of the interaction between the degron and substrate, where the reduction in the affinity led in several cases to increased substrate degradation. The effect was not consistent across all degrons, which might be due to the inherent properties of individual degrons and is likely a balance between affinity and processivity. We expected CDC34 to be more sensitive to the affinity for the substrate by increasing the turnover of E2-Ub complexes in the vicinity of the substrate; however, the effect was not significant. The additional factor that affected degradation were linkers between the coiled coils and the substrate or degron. Here, the effect was more consistent across all degrons, where the longer linkers between CC and the substrate and shorter linkers between the degron and CC both led to an increased substrate degradation. This might be due to the appropriate positioning of the substrate relative to the activated ubiquitin bound to E2 as it has to be in the vicinity of the substrate lysine or nascent Ub chain for the successful conjugation to occur, where the length of the linker might impact the optimal positioning.^2,65,66^ Still, our results demonstrated that the effect of linker length differed if it was on the substrate or the degron side. A possible explanation might be that the shorter linker enabled lower ubiquitination and degradation of a degron construct and the longer linker enabled the more efficient proteasomal entry of the substrate due to the increased unstructured region of the construct, which had previously been shown to have a substantial effect on protein degradation.^64,67^ Although the precise mechanism at play is unclear from our work, it warrants further study, and both the effect of linker length and affinity between the substrate and degron should be taken into account when designing similar systems.

Regulation of protein stability through proteolytically activated degradation is found in natural systems^68^ and engineered into synthetic systems.^69−71^ Here, we show that both the activation and inhibition of degradation can be achieved with E3 ligase-derived degrons. Proteolytic regulation is an attractive strategy for the regulation of cellular processes due to fast ubiquitination and protein degradation, while protein recovery follows the translation rate of each protein. Clinically approved protease inhibitors against NS3/4A, HIV, or other viral proteases could be used as regulators of degradation by replacing the viral proteases used here, as demonstrated by Chung et al. using natural degrons.^71^ Still, the use of chemically induced dimerization using small molecules is a very useful and indeed a popular method of controlling cellular processes. We demonstrated that with the use of a well-established FKBP/FRB/rapamycin CID system, all tested degrons activated degradation of the substrate in a rapamycin-dependent manner, where it was most efficient with Skp1(ΔC111). Recent advances in the design of CIDs will further their use, especially in therapeutic settings, where hypoimmunogenic fragments of human proteins could be particularly beneficial.^41,72^

The value of the synthetic biology toolbox is dependent on its robustness and reliability in different settings. The majority of the SCF-Skp2 E3 ligase-derived degrons were less efficient in degrading membrane proteins but could readily degrade nuclear proteins. To address this limitation in the degradation of membrane proteins, we sourced an additional degron from a different E3 ligase and concatenated it with a best-performing degron from the SCF-Skp2 E3 ligase, mimicking the principle of concatenated transcription factors in a VPR regulator^73^ that become the standard when high efficiency of transcriptional activation is required. This concatenated degron, termed the SSD degron, was able to efficiently degrade nuclear, membrane, and cytosolic proteins in several mammalian cell lines with fast kinetics and efficiency on par with other designed systems of inducible protein degradation.^8,16,46,61,71,74−76^

Our results present an insight into the use of subunits of natural degradation complexes in designed degradation systems, highlight approaches of modulation and control of degradation, and provide a robust method to control the stability of different proteins using CID systems, providing an efficient tool to control biological systems.

## Methods and Materials

### Plasmid Construction

Plasmids were constructed using the Gibson assembly method.^77^ Plasmids and their amino acid sequences are listed in Table S2. CDC34 gene was obtained from pUB6-CDC34-HA (Addgene plasmid #99145) and Cul1 gene from pCMV7.1-3xFLAG-CUL1 (Addgene plasmid #155019), while other E3 ligase motives were synthesized by IDT. sgRNA expression plasmid “pgRNA-humanized” was obtained from Addgene (plasmid no. 44248). The firefly luciferase gene was obtained from pGL4.16 (Promega), and the phRL-TK plasmid (Promega) was used as a transfection control in the dual luciferase assays.

### Cell Culture

The HEK293T and HeLa cell lines (ATCC) were cultured in DMEM medium (Invitrogen) supplemented with 10% FBS (Invitrogen) at 37 °C and 5% CO_2_. CHO and NIH/3T3 cell lines (ATCC) were cultured in DMEM/F12 medium with Glutamax (Invitrogen) supplemented with 10% FBS (Invitrogen) at 37 °C and 5% CO_2_. The Neuro2A cell line (ATCC) was cultured in Optimem media (Invitrogen) supplemented with 10% FBS (Invitrogen) at 37 °C and 5% CO_2_.

### Transfection

For luciferase experiments, 2 × 10^4^ HEK293T, 1 × 10^4^ HeLa, 5 × 10^4^ Neuro2A, 2 × 10^4^ CHO, and 2 × 10^4^ NIH/3T3 cells were seeded into 96-well clear-bottom plates (Corning, type 3610). For cytometer and immunoblotting experiments, 8 × 10^4^ cells were seeded into 24-well flat-bottom plates (TPP). For confocal microscopy, 5 × 10^4^ cells were seeded into an 8-well chamber (Ibidi). Twenty-four hours after seeding at 50–80% confluence, a mixture of plasmid DNA and PEI was added to the cells. PEI was mixed with plasmid DNA at a ratio of 6 μL of PEI for every μg of DNA. The DNA/PEI mixture was incubated for 15 min at room temperature before being added to the cells.

### Rapamycin Stimulation

Rapamycin (Sigma-Aldrich) was dissolved in DMSO at a concentration of 1 mM. Before stimulation, rapamycin in DMSO was diluted in media at a 10× final concentration and an appropriate volume of rapamycin/media solution was added to the cell media to achieve a 1× final concentration.

### Luciferase Assay

Cells were harvested 48 h after transfection (or for kinetic experiments at indicated time points) and lysed using 25 μL of Passive lysis buffer (Biotium). Firefly and Renilla luciferase activity was measured in cell lysate using the dual luciferase assay (Promega), and luminescence was measured on a Centro microplate luminometer (Berthold Technologies). Relative luciferase units (RLU) were calculated by normalizing each sample’s luciferase signal to the renilla signal of the same sample.

### Flow Cytometry

Forty-eight hours post-transfection cells were harvested by removing cell media and resuspending in PBS. Flow cytometry analysis was performed on an Aurora flow cytometer (Cytek Biosciences) by using cells transfected with an empty vector (pcDNA), pcDNA-BFP, and pcDNA-mCit as controls for unmixing. Representative cell gating is presented in Figure S12.

### Confocal Microscopy

Two days after transfection, microscopy images were acquired using a Leica TCS SP5 inverted laser-scanning microscope on a Leica DMI 6000 CS module equipped with a HCX PL Fluotar L ×20, numerical aperture 0.4 (Leica Microsystems). For mCitrine excitation, a 514 nm laser line of a 100 mW argon laser was used, and emitted light was detected between 530 and 550 nm. For BFP excitation, a 50 mW 405 nm diode laser was used, and the emitted light was detected between 420 and 460 nm. Leica LAS AF software was used for image acquisition.

### Immunoblotting

Forty-eight hours after transfection, cells were washed with PBS and resuspended in 200 μL of Passive lysis buffer (Biotium) and lysed for 30 min on ice. After, the lysates were centrifuged for 5 min at 14,200 rpm on a tabletop centrifuge to remove cell debris. The total protein concentration was measured in the supernatant using a BCA assay (Sigma-Aldrich). Then, 30 μg of each sample was denatured by incubating the sample at 95 °C for 5 min with SDS. Samples were loaded on 10% SDS-PAGE gels and separated at 200 V for 40 min. Proteins were transferred to a nitrocellulose membrane using an iBlot 2 gel transfer device (Invitrogen) according to the manufacturer’s protocol. Membrane blocking, washing, and antibody binding were performed using the iBind Flex Western device (Invitrogen) according to the manufacturer’s protocol. Primary antibodies were rabbit Anti-HA (Merck, diluted 1:1000) and mouse β-actin (Cell Signaling Technology, diluted 1:5000). Secondary antibodies used were goat antirabbit (Abcam, diluted 1:4000) and goat antimouse (Jackson ImmunoResearch, diluted 1:3000). A signal was developed with a SuperSignal West Pico (Thermo Fischer Scientific) substrate according to manufacturer’s protocol, and blots were visualized on a G-box device (Syngene).

### Software and Statistical Analysis

A two-sided unpaired *t* test was performed using GraphPad Prism 8.4.3 software. *P* values on the graph are summarized as follows: ****P < 0.0001; ***P < 0.001; **P < 0.01; *P < 0.05; not significant (n.s.) *P* > 0.05).

Cytometry data was analyzed on FlowJo software, version 10.4.

Gel image band intensity after immunoblot membrane visualization was quantified by using ImageJ software.

## Supporting Information Available

Degron sequence information, additional experiments with proteasome inhibitors, additional experiments on the effects of rapamycin or DMSO, gating procedure data for flow cytometry experiments, data on all plasmid transfection mixtures and sequences of proteins expressed in this study (PDF)
