# Correction to Subunits of E3 Ligase Complex as Degrons for Efficient Degradation of Cytosolic, Nuclear and Membrane Proteins

**DOI:** 10.1021/acssynbio.4c00257

**Published:** 2024-04-28

**Authors:** Anže Verbič, Tina Lebar, Arne Praznik, Roman Jerala

**Affiliations:** † Department of Synthetic Biology and Immunology, National Institute of Chemistry, 1000 Ljubljana, Slovenia; § Interdisciplinary Doctoral Study of Biomedicine, Medical Faculty, University of Ljubljana, 1000 Ljubljana, Slovenia

An author affiliation was inadvertently omitted in the original version of the paper. The author list should appear as it does in this Addition and Correction.

