# Supplementary material for: Subunits of an E3 Ligase Complex as Degrons for Efficient Degradation of Cytosolic, Nuclear, and Membrane Proteins
Source: ACS Synth Biol. 2024 Feb 26;13(3):792–803. doi: 10.1021/acssynbio.3c00588 (PMC10949250; doi:10.1021/acssynbio.3c00588)
Supplement: Supplementary file 1 — sb3c00588_si_001.pdf [file sb3c00588_si_001.pdf]

Supporting information

Subunits of E3 ligase complex as degrons for efficient degradation of cytosolic, nuclear and membrane proteins

Anže Verbič[1], Tina Lebar[1,2], Arne Praznik[1], Roman Jerala[1,*]

[1]Department of Synthetic Biology and Immunology, National Institute of Chemistry, 1000 Ljubljana, Slovenia

[2]current address: Wyss Institute for Biologically Inspired Engineering, Harvard University, Boston, MA 02215, USA

*corresponding author, e-mail: roman.jerala@ki.si

Contents

Note S1: Design of degrons based on E3 ligase 2

Figure S1 – Skp2 aminoacid sequence and interaction data 3

Figure S2 – Skp1 aminoacid sequence and interaction data 4

Figure S3 – Cul1 aminoacid sequence and interaction data 5

Figure S4 – Rbx1 aminoacid sequence and interaction data 6

Figure S5 – SOCS2 sequence and interaction data 7

Figure S6 – Degradation potency of SCF-Skp2 E3 ligese derived degrons in direct fusion with firefly luciferase substrate is proteasome dependant. 8

Figure S7 - Western blot of coiled-coil mediated degradation of luciferase substrate. 9

Figure S8 - Degradation potency of SCF-Skp2 E3 ligese derived degrons in coiled-coil mediated interaction with firefly luciferase substrate is proteasome dependant. 10

Figure S9 – Heat map data on the effect of linker length between CC dimer and both the substrate and degron. 11

Figure S10 – Effect of rapamycin and DMSO on the expression of luciferase reporter substrates..12

Figure S11 – CAR proteins translocate to the plasma membrane in HEK293T cells. 13

Figure S12 – Representative gating procedure for flow cytometry experiments. 14

Table S1 – Plasmid transfection mixtures performed in this study 15

Table S2 – Amino acid sequences of constructs used in this study 16

References 25

Note S1: Design of degrons based on E3 ligase

The design of degrons from on SCF-Skp2 E3 ligase was based on previously published data on structure of Cul1-Rbx1-Skp1- F box^{Skp2} complex and interactions between its constituent proteins. [1,2] Design of SOCSbox degron is based on previously published data on structure of SOCS2-ElonginC-ElonginB E3 ligase complex. [3] Interaction data is summarized in figures S1-S6.

Skp2 **F-box** is based on a 69 aminoacid long F-box motif from Skp2. It consists of aminoacid residues 101-169 of human Skp2 between and including L1 and H4 domains. Sequence and interaction data is presented in figure S1. [2]

With **Skp1**, we tested the full-length 162 aminoacid long human Skp1 protein. Skp1 binds Skp2 with C-terminal H5, H6, H7 and H8 helices. [2] Skp1 mutants were designed to not include these helices to prevent binding to cellular F-box proteins. H8 and H7 were removed in **Skp1(ΔC131)**, H8, H7 and H6 in **Skp1(ΔC111)** and H8, H7,H6, and H5 in **Skp1(ΔC94)**. Number in the mutant name represents the amino acid location in the Skp1 where the C-terminal deletion is made. Sequence and interaction data is presented in figure S2.

Cul1(ΔN501) is based on 275 C-terminal aminoacids of human Cul1 protein, which contain Rbx1 binding motifs. [1] Sequence and interaction data is presented in figure S3.

With **Rbx1**, we tested the whole 107 aminoacid long human Rbx1 protein. The **Rbx1(ΔN36)** mutant is missing 36 N-terminal aminoacids, that contain Cul1 binding motif. Rbx1(ΔN36) still contains C-terminal RING motif, which binds cellular E2 proteins. Such mutant would not be able to bind cellular Cul1 proteins but still be able to bind E2 proteins and potentially act as a degron. Sequence and interaction data is presented in figure S4.

SOCSbox consists of 41 C-terminal aminoacids of human SOCS2 protein containing H1, H2 and H3 alfa helices, which interact with ElonginB and ElonginC from the SOCS2-ElonginC-ElonginB E3 ligase complex. [3] Sequence and interaction data is presented in figure S5.

```
RENFPGVSWDSLPDELLLGIFSCLCLPELLKVSGVCKRWYRLASDESLWQTLDLTGKNLH 161
PDVTGRLLSQGVIAFRCPRSFMDQPLAEHFSPFRVQHMDLSNSVIEVSTLHGILSQCSKL 221
QNLSLEGLRLSDPIVNTLAKNSNLVRLNLSGCSGFSEFALQTLLSSCSRLDELNLSWCFD 281
FTEKHVQVAVAHVSETITQLNLSGYRKNLQKSDLSTLVRRCPNLVHLDLSDSVMLKNDCF 341
QEFFQLNYLQHLSLSRCYDIIPETLLELGEIPTLKTLQVFGIVPDGTLQLLKEALPHLQI 401
NCSHFTTIARPTIGNKKNQEIWGIKCRLTLQKPSCL
```

Skp1-binding residues

Figure S1 – Skp2 aminoacid sequence and interaction data
Amino acid sequence of human Skp2. Amino acid residues in red bind Skp1. Data on interacting amino acid residues is based on previously published reports. Aminoacid positions are annotated to human Skp2, with sequence before aminoacid 101 not shown. [2]

PSIKLQSSDGEIFEVDVEIAKQSVTIKTMLEDLGMDDEGDDDPVPLPNVNAAILKKVIQW 60

CTHHKDDPPPPEDDENKEKRTDDIPVWDQEFLKVDQGTLFELILAANYLDIKGLLDVTCK 120

TVANMIKGKTPEEIRKTFNIKNDFTEEEEAQVRKENQWCEEK

Skp2-binding residues

Cul1-binding residues

Figure S2 – Skp1 aminoacid sequence and interaction data

Amino acid sequence of human Skp1. Amino acid residues in violet bind Skp2. Data on interacting amino acid residues is based on previously published reports. [2]

```
SSTRSQNPHGLKQIGLDQIWDDLRAGIQQVYTRQSMAKSRYMELYTHVYNYCTSVHQSNQ  60
ARGAGVPPSKSKKGQTPGGAQFVGLELYKRLKEFLKNYLTNLLKDGEDLMDESVLKFYTQ 120
QWEDYRFSSKVLNGICAYLNRHWVRRECDEGRKGIYEIYSLALVTWRDCLFRPLNKQVTN 180
AVLKLIEKERNGETINTRLISGVVQSYVELGLNEDDAFAKGPTLTVYKESFESQFLADTE 240
RFYTRESTEFLQQNPVTEYMKKAEARLLEEQRRVQVYLHESTQDELARKCEQVLIEKHLE 300
IFHTEFQNLLDADKNEDLGRMYNLVSRIQDGLGELKKLLETHIHNQGLAAIEKCGEAALN 360
DPKMYVQTVLDVHKKYNALVMSAFNNDAGFVAALDKACGRFINNNAVTKMAQSSSKSPEL 420
LARYCDSLLKKSSKNPEEAELEDTLNQVMVVFKYIEDKDVFQKFYAKMLAKRLVHQNSAS 480
DDAEASMISKLKQACGFEYTSKLQRMFQDIGVSKDLNEQFKKHLTNSEPLDLDFSIQVLS 540
SGSWPFQQSCTFALPSELERSYQRFTAFYASRHSGRKLTWLYQLSKGELVTNCFKNRYTL 600
QASTFQMAILLQYNTEDAYTVQQLTDSTQIKMDILAQVLQILLKSKLLVLEDENANVDEV 660
ELKPDTLIKLYLGYKNKKLRVNINVPMKTEQKQEQETTHKNIEEDRKLLIQAAIVRIMKM 720
RKVLKHQQLLGEVLTQLSSRFKPRVPVIKKCIDILIEKEYLERVDGEKDTYSYLA
```

Skp1/F-box-binding residues

Rbx1-binding residues

Figure S3 – Cul1 aminoacid sequence and interaction data
Amino acid sequence of human Cul1. Amino acid residues in yellow and green bind Skp1/F-box and Rbx1 respectively. Data on interacting amino acid residues is based on previously published reports. [1]

AAAMDVDTPSGTNSGAGKKRFEVKKWNAVALWAWDIVVDNCAICRNHIMDLCIECQANQA 60

SATSEECTVAWGVCNHAFHFHCISRWLKTRQVCPLDNREWEFQKYGH

Cul1-binding residues

Zinc-chelating residues

Figure S4 – Rbx1 aminoacid sequence and interaction data

Amino acid sequence of human Rbx1. Amino acid residues in red bind Cul1 while amino acid residues in blue form are a part of RING motif and form a Zinc-binding site. Data on interacting amino acid residues is based on previously published reports. [1]

MTLRCLEPSGNGGEGTRSQWGTAGSAEEPSPQAARLAKALRELGQTGWYWGSMTVNEAKE 60

KLKEAPEGTFLIRDSSHSDYLLTISVKTSAGPTNLRIEYQDGKFRLDSIICVKSKLKQFD 120

SVVHLIDYYVQMCKDKRTGPEAPRNGTVHLYLTKPLYTSAPSLQHLCRLTINKCTGAIWG 180

LPLPTRLKDYLEEYKFQV 198

ElonginC/B-binding residues

Figure S5 – SOCS2 sequence and interaction data

Amino acid sequence of human SOCS2 protein. Amino acid residues in green bind ElonginC/B. Data on interacting amino acid residues is based on previously published reports. [3]

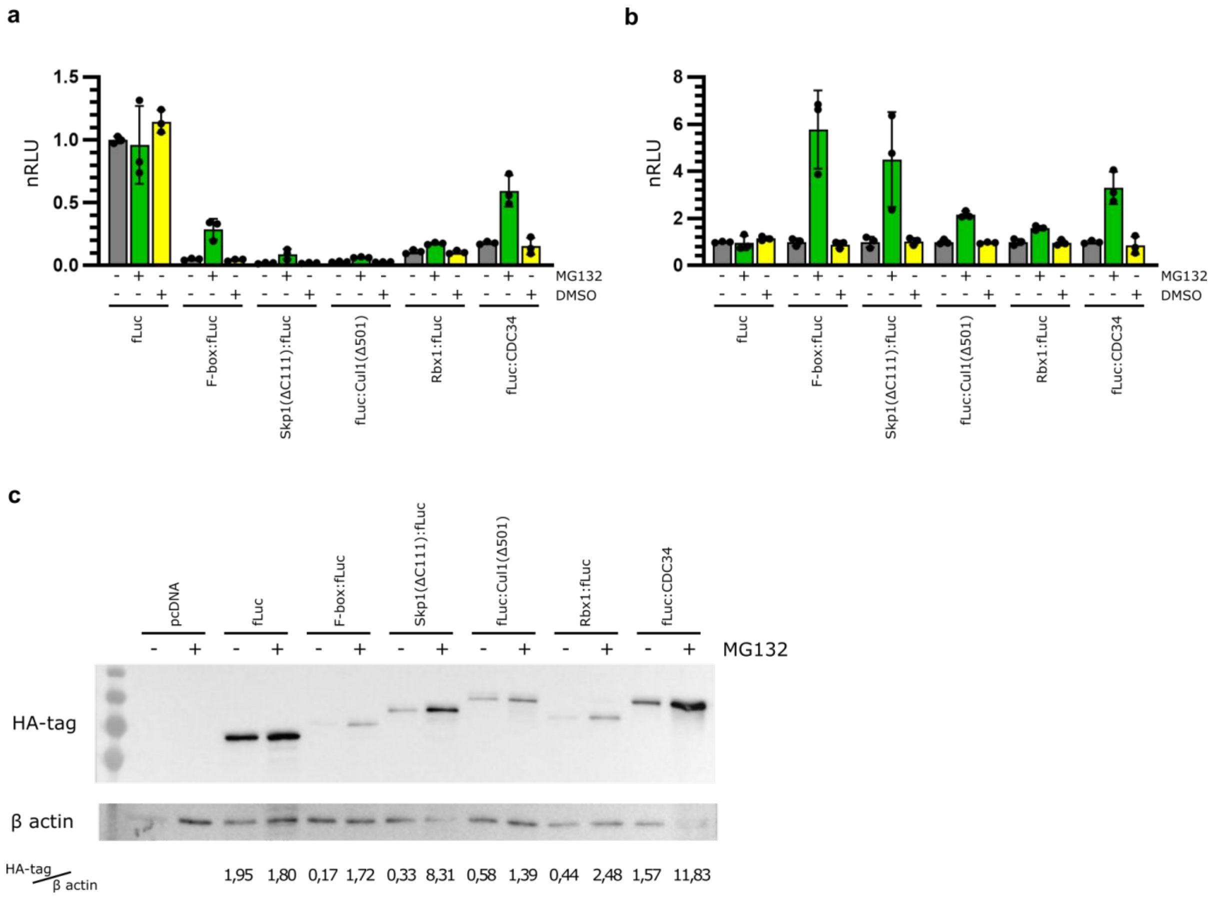

Figure S6 – Degradation potency of SCF-Skp2 E3 ligese derived degrons in direct fusion with firefly luciferase substrate is proteasome dependant.

a Inhibition of substrate degradation by MG132 proteasome inhibitor. Plasmids expressing luciferase substrate in fusion with best performing degrons were cotransfected into HEK293T cells. 24h after transfection MG132 proteasome inhibitor in DMSO at final concentration of 3 µM or DMSO at the same v/v ratio were added in treated wells. Luciferase activity was measured 48h post-transfection. Values represent the mean ± SD of three cell cultures experiments and are normalized to the expression of luciferase without degron fusion and untreated with MG132 or DMSO. **b** Representation of the same data as in panel a with the data for each luciferase and degron fusion normalized to the untreated condition. **c** Western blot analysis of expression of luciferase substrate in fusion with best performing degrons in the presence and absence of MG132. Plasmids expressing luciferase substrate fusion proteins were transfected into HEK293T cells. 24h post transfection MG132 proteasome inhibitor in DMSO at final concentration of 3 µM were added in treated wells. Immunoblotting is described in the main body of the paper, transfection plasmid mixtures are listed in Table S1. Gel image band intensity was quantified using ImageJ software. Band intensity values are normalized to the intensity of beta-actin band.

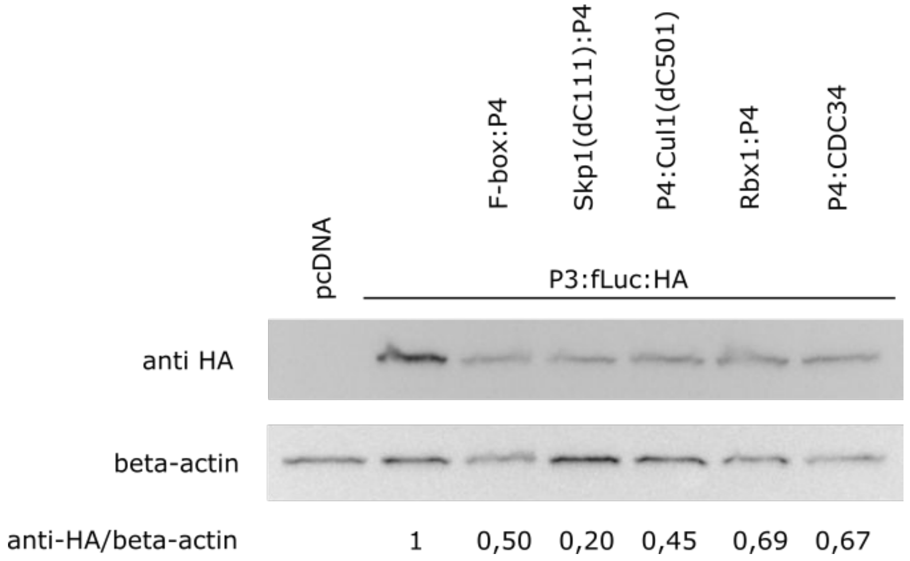

Figure S7 - Western blot of coiled-coil mediated degradation of luciferase substrate.

HA-tagged firefly luciferase substrate protein fused with the P3 CC-forming peptide was cotransfected with individual degrons fused with P4 partner CC-forming peptide. Transfection and immunoblotting is described in the main body of the paper, transfection plasmid mixtures are listed in Table S1. Gel image band intensity was quantified using ImageJ software. All band intensity values are normalized to the intensity of beta-actin band, ratios of anti-HA/beta-actin are normalized to the anti-HA/beta-actin ratio of firefly luciferase without cotransfected degrons.

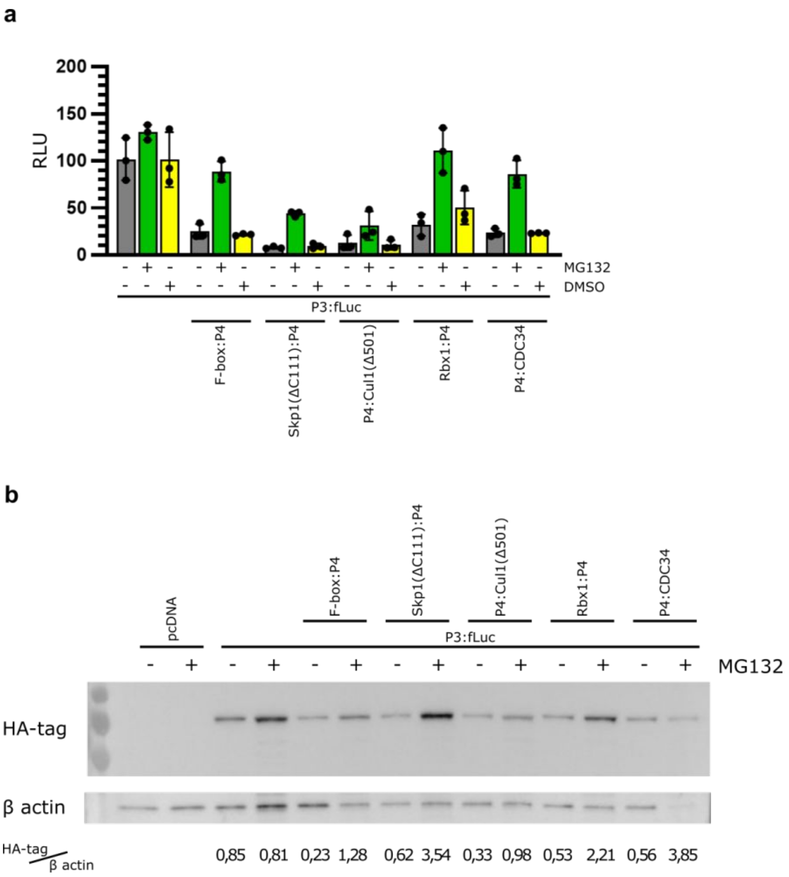

Figure S8 - Degradation potency of SCF-Skp2 E3 ligese derived degrons in coiled-coil mediated interaction with firefly luciferase substrate is proteasome dependant.

a Inhibition of coiled-coil mediated substrate degradation by MG132 proteasome inhibitor. Plasmids expressing luciferase substrate in fusion with P3 coil-forming peptide and best performing degrons in fusion with P4 coil-forming peptide were cotransfected into HEK293T cells. 24h after transfection MG132 proteasome inhibitor in DMSO at final concentration of 3 µM or DMSO at the same v/v ratio were added in treated wells. Luciferase activity was measured 48h post-transfection. Values represent the mean ± SD of three cell cultures experiments and are normalized to the expression of luciferase without cotransfected degron constructs and untreated with MG132 or DMSO. **b** Western blot analysis of expression of luciferase substrate in fusion with P4 coil-forming peptide in cotransfection with best performing degrons in fusion with P4 coil-forming peptide in the presence and absence of MG132. Plasmids expressing the described proteins were transfected into HEK293T cells. 24h post transfection MG132 proteasome inhibitor in DMSO at final concentration of 3 µM were added in treated wells. Immunoblotting is described in the main body of the paper, transfection plasmid mixtures are listed in Table S1. Gel image band intensity was quantified using ImageJ software. Band intensity values are normalized to the intensity of beta-actin band.

F-box

degron-CC linker length	gsg			gs10			gs30		
substrate-CC linker length	gs3	gs10	gs30	gs3	gs10	gs30	gs3	gs10	gs30
	0,458	0,479	0,187	0,474	0,483	0,258	0,582737	0,518743	0,375997
	0,573	0,519	0,204	0,424	0,495	0,259	0,720894	0,561553	0,359058
	0,456	0,487	0,221	0,456	0,475	0,216	0,695452	0,539995	0,390356
average	0,495667	0,495	0,204	0,451333	0,484333	0,244333	0,666361	0,540097	0,375137
stdev	0,054689	0,017282	0,01388	0,020677	0,008219	0,020039	0,060037	0,017477	0,012792

significance of substrate-CC linker length	P value
S gsg D gs30, S gsg D gsg	0,041
S gs10 D gs30, S gs10 D gsg	0,0604
S gs30 D gs30, S gs30 D gsg	0,0002
**significance of degron-CC linker length**	
S gsg D gsg, S gs30 D gsg	0,0019
S gsg D gs10, S gs30 D gs10	0,0005
S gsg D gs30, S gs30 D gs30	0,0026

Skp1(ΔC111)

degron-CC linker length	gsg			gs10			gs30		
substrate-CC linker length	gs3	gs10	gs30	gs3	gs10	gs30	gs3	gs10	gs30
	0,2219	0,2219	0,0861	0,287	0,2044	0,1281	1,143252	0,552972	0,403025
	0,2394	0,2394	0,1064	0,294	0,1883	0,1414	1,14045	0,596028	0,384902
	0,2303	0,2303	0,1057	0,2548	0,2198	0,1022	0,907137	0,595613	0,322539
average	0,230533	0,230533	0,0994	0,2786	0,204167	0,1239	1,063613	0,581538	0,370155
stdev	0,007146	0,007146	0,009409	0,01707	0,012861	0,016277	0,110651	0,0202	0,034473

significance of substrate-CC linker length	P value
S gsg D gs30, S gsg D gsg	0,0004
S gs10 D gs30, S gs10 D gsg	<0,0001
S gs30 D gs30, S gs30 D gsg	0,0004
**significance of degron-CC linker length**	
S gsg D gsg, S gs30 D gsg	<0,0001
S gsg D gs10, S gs30 D gs10	0,0008
S gsg D gs30, S gs30 D gs30	0,0011

Cul1(ΔC501)

degron-CC linker length	gsg			gs10			gs30		
substrate-CC linker length	gs3	gs10	gs30	gs3	gs10	gs30	gs3	gs10	gs30
	0,577	0,459	0,249	0,707	0,599	0,348	0,520933	0,493493	0,417821
	0,628	0,5	0,266	0,717	0,631	0,344	0,581307	0,502099	0,402869
	0,618	0,436	0,267	0,621	0,567	0,32	0,541608	0,512068	0,407542
average	0,607667	0,465	0,260667	0,681667	0,599	0,337333	0,54795	0,502554	0,409411
stdev	0,022066	0,02647	0,00826	0,043092	0,026128	0,012365	0,025052	0,00759	0,006245

significance of substrate-CC linker length	P value
S gsg D gs30, S gsg D gsg	0,0647
S gs10 D gs30, S gs10 D gsg	0,126
S gs30 D gs30, S gs30 D gsg	<0,0001
**significance of degron-CC linker length**	
S gsg D gsg, S gs30 D gsg	<0,0001
S gsg D gs10, S gs30 D gs10	0,0004
S gsg D gs30, S gs30 D gs30	0,0016

Rbx1

degron-CC linker length	gsg			gs10			gs30		
substrate-CC linker length	gs3	gs10	gs30	gs3	gs10	gs30	gs3	gs10	gs30
	0,379971	0,335266	0,137406	0,311564	0,371182	0,25246	1,016304	0,768634	0,531338
	0,290494	0,35218	0,161403	0,267931	0,341668	0,242766	0,970645	0,7592	0,501049
	0,26896	0,325954	0,168595	0,323149	0,319279	0,181551	0,996569	0,727466	0,506288
average	0,313142	0,3378	0,155801	0,300881	0,344043	0,225592	0,994506	0,751767	0,512892
stdev	0,048066	0,010856	0,013335	0,023775	0,021256	0,031392	0,018697	0,01761	0,013218

significance of substrate-CC linker length	P value
S gsg D gs30, S gsg D gsg	0,0112
S gs10 D gs30, S gs10 D gsg	0,0539
S gs30 D gs30, S gs30 D gsg	<0,0001
**significance of degron-CC linker length**	
S gsg D gsg, S gs30 D gsg	0,0112
S gsg D gs10, S gs30 D gs10	0,0539
S gsg D gs30, S gs30 D gs30	<0,0001

CDC34

degron-CC linker length	gsg			gs10			gs30		
substrate-CC linker length	gs3	gs10	gs30	gs3	gs10	gs30	gs3	gs10	gs30
	0,385177	0,173989	0,167695	0,341831	0,268818	0,226662	0,478725	0,339334	0,361445
	0,281356	0,208071	0,228083	0,309699	0,24507	0,266379	0,481758	0,317241	0,379403
	0,275956	0,189119	0,137392	0,305489	0,260556	0,201441	0,473233	0,398809	0,388263
average	0,314163	0,190393	0,177723	0,319006	0,258148	0,231494	0,477905	0,351795	0,37637
stdev	0,050263	0,013943	0,037697	0,016231	0,009843	0,02673	0,003528	0,034446	0,011157

significance of substrate-CC linker length	P value
S gsg D gs30, S gsg D gsg	0,0101
S gs10 D gs30, S gs10 D gsg	0,0036
S gs30 D gs30, S gs30 D gsg	0,002
**significance of degron-CC linker length**	
S gsg D gsg, S gs30 D gsg	0,0373
S gsg D gs10, S gs30 D gs10	0,0167
S gsg D gs30, S gs30 D gs30	0,0003

Figure S9 – Heat map data on the effect of linker length between CC dimer and both the substrate and degron. Values in Fig. 2c in the main body of the paper represent mean values of values presented here. Statistical analysis was conducted by performing two-sided unpaired t-test and is presented for each individual degron for selected data, indicated on the right, comparing both CC-degron and CC-substrate degradation. S – linker between CC and substrate, D – linker between CC and degron.

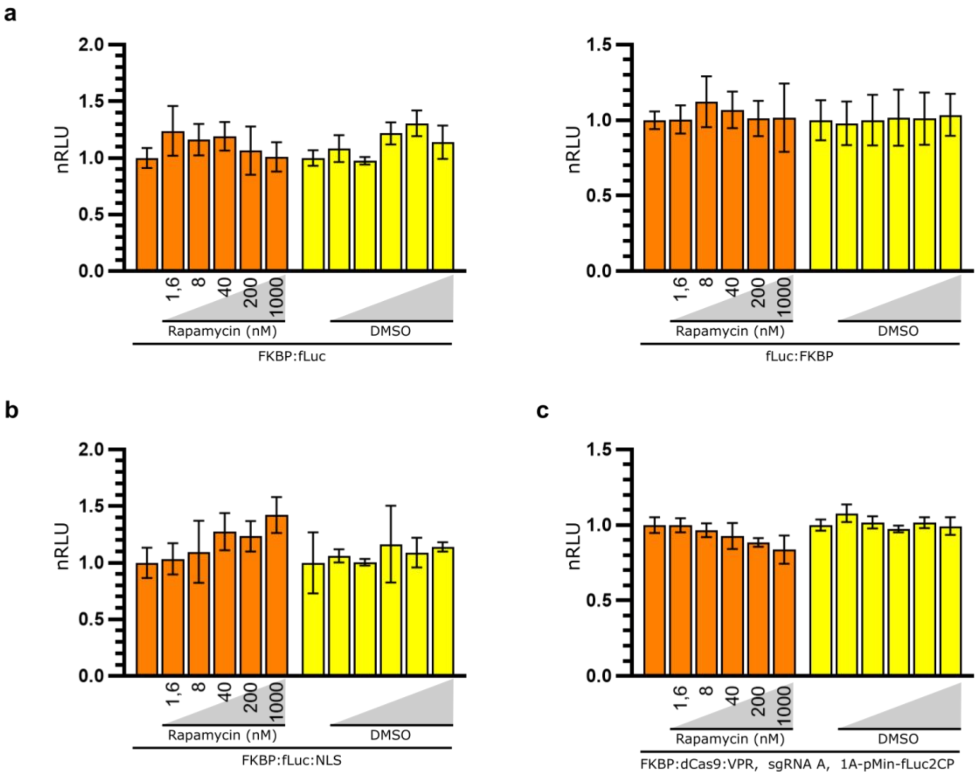

Figure S10 – Effect of rapamycin and DMSO on the expression of luciferase reporter substrates.

Rapamycin or DMSO might effect the expression of luciferase substrates used in this study with a mechanism other than degradation by dimerization of substrate with degron, as we proposed. Here we titrated rapamycin and DMSO (at the same v/v ratio as rapamycin) to HEK293T cells transfected with all luciferase based substrate reporter proteins used in this study: FKBP:fLuc and fLuc:FKBP (**a**), FKBP:fLuc:NLS (**b**) and firefly luciferase reporter gene under minimal promotor in cotransfection with dCas and sgRNA (**c**). Plasmids expressing the described proteins were cotransfected into HEK293T cells. Rapamycin or DMSO were added to wells 24h post-transfection and luciferase activity was measured 48h post-transfection.

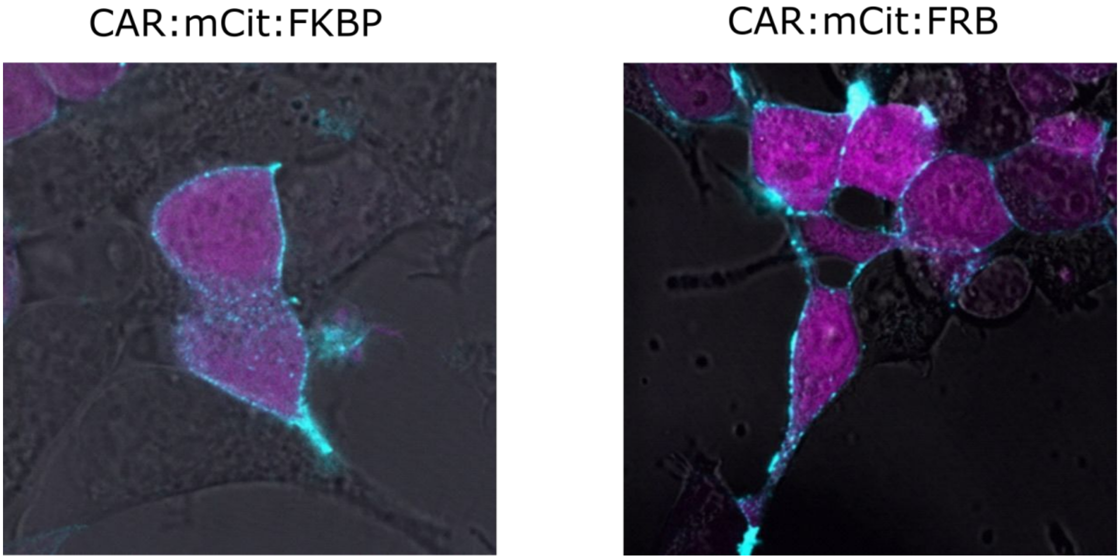

Figure S11 – CAR proteins translocate to the plasma membrane in HEK293T cells. HEK293T cells were transfected with CAR:mCit:FKBP or CAR:mCit:FRB and BFP. Images were acquired 48 hours after transfection according to the methods described in the main body of this paper. mCitrine emission (530-550 nm) is here colored in cyan and BFP emission (420-460 nm) in magenta. As expected BFP is localized in the cytosol while CAR constructs localize to the plasma membrane.

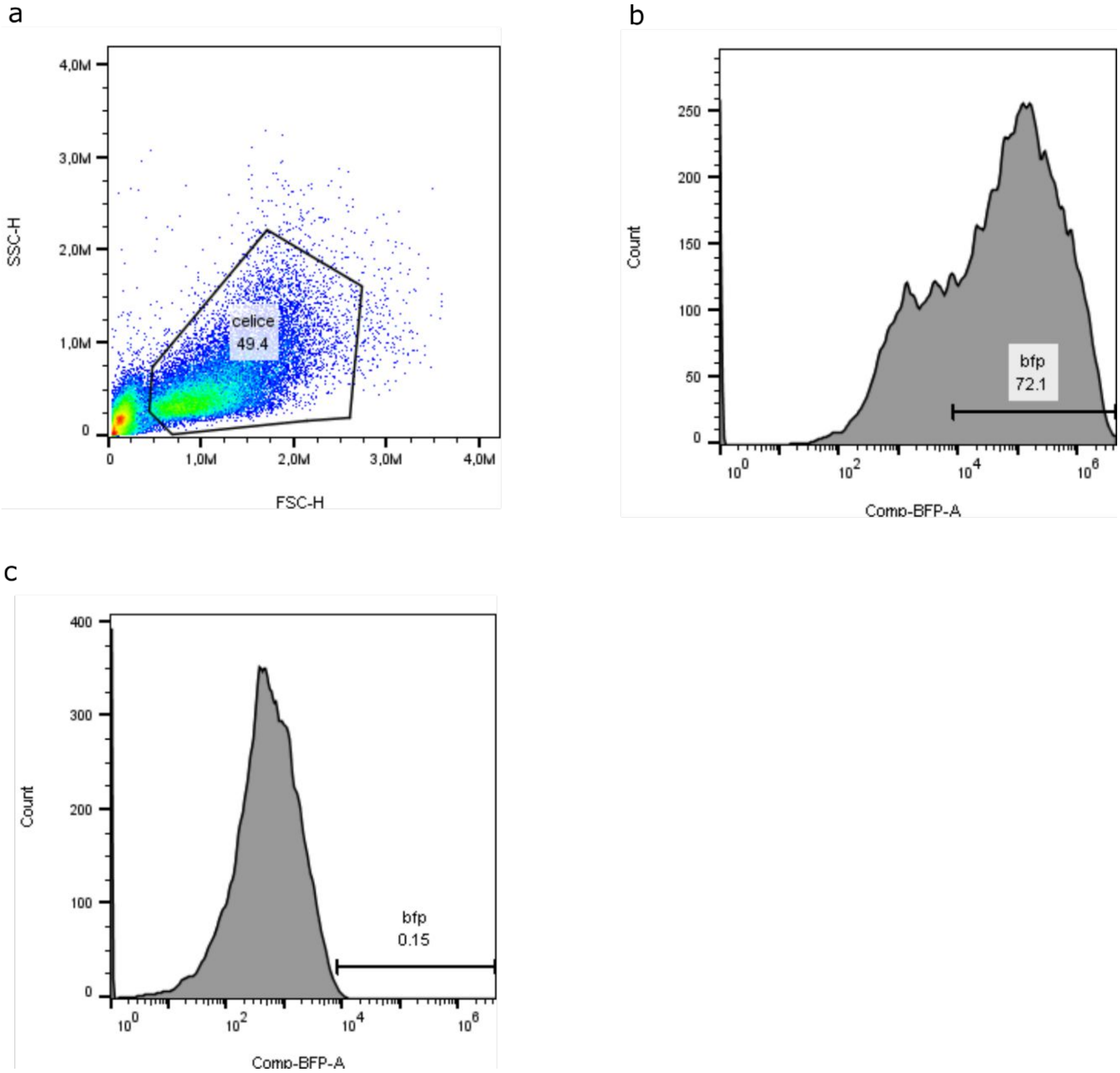

Figure S12 – Representative gating procedure for flow cytometry experiments.

Data acquired using Aurora flow cytometer (Cytek Biosciences) were analysed using FlowJo software version 10.4. Event data was first gated to exclude non-cell events (panel **a**) and then gated again to include events with sufficient signal of BFP (panel **b**). BFP gating on untransfected cells is shown in panel **c**.

Table S1 – Plasmid transfection mixtures performed in this study

Figure	Description of plasmid transfection mixture
1c	10 ng of each construct described in the figure, 10 ng Renilla plasmid, empty pcDNA to a final amount of 200 ng plasmid DNA per well
1d	Same as in Fig. 1c
2a	10 ng of P3:fLuc construct, amounts of P4:degron construct are stated in the figure, 10 ng Renilla plasmid, empty pcDNA to a final amount of 200 ng plasmid DNA per well
2b	10 ng of P3:fLuc construct, 50 ng CC:degron construct, 10 ng Renilla plasmid, empty pcDNA to a final amount of 200 ng plasmid DNA per well
2c	10 ng of each construct described in the figure, 10 ng Renilla plasmid, empty pcDNA to a final amount of 200 ng plasmid DNA per well
3a	10 ng of AP4:TEVs:P3:fLuc, 75 ng of each P4:degron construct, amounts of TEVp are stated in the figure, 10 ng Renilla plasmid, empty pcDNA to a final amount of 200 ng plasmid DNA per well
3b	10 ng of each luciferaze and degron fusion protein construct, amounts of TEVp are stated in the figure, 10 ng Renilla plasmid, empty pcDNA to a final amount of 200 ng plasmid DNA per well
3c	10 ng of luciferaze-FKBP fusion construct, 100 ng of each FRB-degron fusion protein construct, 10 ng Renilla plasmid, empty pcDNA to a final amount of 200 ng plasmid DNA per well
4b	50 ng of CAR-mCit-FRB construct, 400 ng of degron-FKBP construct, 100 ng of BFP, empty pcDNA to a final amount of 800 ng plasmid DNA per well
4c	10 ng of luciferaze-FKBP fusion construct, 100 ng of each FRB-degron fusion protein construct, 10 ng Renilla plasmid, empty pcDNA to a final amount of 200 ng plasmid DNA per well
4d	50 ng of 1A-pMin-fLuc2CP reporter plasmid, 5 ng of FKBP:dCas9:VPR, 25 ng of sgRNA A, 100 ng of each FRB-degron fusion protein construct, 10 ng Renilla plasmid, empty pcDNA to a final amount of 200 ng plasmid DNA per well
5b	*Left and right graph* - 10 ng of luciferaze-FKBP fusion construct, 100 ng of each FRB-degron fusion protein construct, 10 ng Renilla plasmid, empty pcDNA to a final amount of 200 ng plasmid DNA per well<br>*Middle graph* - 50 ng of CAR-mCit-FRB construct, 400 ng of degron-FKBP construct, 100 ng of BFP, empty pcDNA to a final amount of 800 ng plasmid DNA per well
5c	10 ng of FKBP:fLuc fusion construct, 100 ng of each FRB:SSD fusion protein construct, 10 ng Renilla plasmid, empty pcDNA to a final amount of 200 ng plasmid DNA per well
5d	10 ng of FKBP:fLuc fusion construct, 100 ng of each FRB:SSD fusion protein construct, 10 ng Renilla plasmid, empty pcDNA to a final amount of 200 ng plasmid DNA per well
S6a	10 ng of each construct described in the figure, 10 ng Renilla plasmid, empty pcDNA to a final amount of 200 ng plasmid DNA per well
S6b	Same as in S6a
S6c	50 ng of each luciferase substrate-degron fusion protein construct, empty pcDNA to a final amount of 1600 ng plasmid DNA per well
S7	50 ng of P3:fluc:HA construct, 500 ng of each P4-degrons fusion protein construct, empty pcDNA to a final amount of 1600 ng plasmid DNA per well
S8a	10 ng of P3:fluc, 100 ng of each degron construct described in the figure, 10 ng Renilla plasmid, empty pcDNA to a final amount of 200 ng plasmid DNA per well

S8b	50 ng of P3:fluc:HA construct, 500 ng of each P4-degrons fusion protein construct, empty pcDNA to a final amount of 1600 ng plasmid DNA per well
S10	10 ng of each construct described in the figure, 10 ng Renilla plasmid, empty pcDNA to a final amount of 200 ng plasmid DNA per well
S11	50 ng of CAR:mCit:FKBP or CAR:mCit:FRB construct, 100 ng of BFP plasmid, empty pcDNA to a final amount of 400 ng plasmid DNA per well

Table S2 – Amino acid sequences of constructs used in this study

Fig.	Short name	Long name + amino acid sequence
1c	fLuc	pcDNA3.1-fLuc
		EDAKNIKKGPAPFYPLEDGTAGEQLHKAMKRYALVPGTIAFTDAHIEVDITYAEYFEMSVRLAEAM<br>KRYGLNTNHRIVVCSENSLQFFMPVLGALFIGVAVAPANDIYNERELLNSMGISQPTVVFVSKKGL<br>QKILNVQKKLPIIQKIIIMDSKTDYQGFQSMYTFVTSHLPPGFNEYDFVPESFDRDKTIALIMNSS<br>GSTGLPKGVALPHRTACVRFSHARDPIFGNQIIPDTAILSVVPFHHGFGMFTTLGYLICGFRVVLM<br>YRFEEELFLRSLQDYKIQSALLVPTLFSFFAKSTLIDKYDLSNLHEIASGGAPLSKEVGEAVAKRF<br>HLPGIRQGYGLTETTSAILITPEGDDKPGAVGKVVPFFEAKVVDLDTGKTLGVNQRGELCVRGPMI<br>MSGYVNNPEATNALIDKDGWLHSGDIAYWDEDEHFFIVDRLKSLIKYKGYQVAPAELESILLQHPN<br>IFDAGVAGLPDDDAGELPAAVVVLEHGKTMTEKEIVDYVASQVTTAKKLRGGVVFVDEVPKGLTGK<br>LDARKIREILIKAKKGGKIAVNS
	F-box:fLuc	pcDNA3.1-f-box:gs10:fluc
		RENFPGVSWDSLPDELLLGIFSCLCLPELLKVSGVCKRWYRLASDESLWQTLDLTGKNLHPDVTGR<br>LLSGGSGGGSGGSEDAKNIKKGPAPFYPLEDGTAGEQLHKAMKRYALVPGTIAFTDAHIEVDITYA<br>EYFEMSVRLAEAMKRYGLNTNHRIVVCSENSLQFFMPVLGALFIGVAVAPANDIYNERELLNSMGI<br>SQPTVVFVSKKGLQKILNVQKKLPIIQKIIIMDSKTDYQGFQSMYTFVTSHLPPGFNEYDFVPESF<br>DRDKTIALIMNSSGSTGLPKGVALPHRTACVRFSHARDPIFGNQIIPDTAILSVVPFHHGFGMFTT<br>LGYLICGFRVVLMYRFEEELFLRSLQDYKIQSALLVPTLFSFFAKSTLIDKYDLSNLHEIASGGAP<br>LSKEVGEAVAKRFHLPGIRQGYGLTETTSAILITPEGDDKPGAVGKVVPFFEAKVVDLDTGKTLGV<br>NQRGELCVRGPMIMSGYVNNPEATNALIDKDGWLHSGDIAYWDEDEHFFIVDRLKSLIKYKGYQVA<br>PAELESILLQHPNIFDAGVAGLPDDDAGELPAAVVVLEHGKTMTEKEIVDYVASQVTTAKKLRGGV<br>VFVDEVPKGLTGKLDARKIREILIKAKKGGKIAVNS
	Skp1:fLuc	pcDNA3.1-Skp1:gs10:fluc
		PSIKLQSSDGEIFEVDVEIAKQSVTIKTMLEDLGMDDEGDDDPVPLPNVNAAILKKVIQWCTHHKD<br>DPPPPEDDENKEKRTDDIPVWDQEFLKVDQGTLFELILAANYLDIKGLLDVTCKTVANMIKGKTPE<br>EIRKTFNIKNDFTEEEEAQVRKENQWCEEKGGSGGGSGGSEDAKNIKKGPAPFYPLEDGTAGEQLH<br>KAMKRYALVPGTIAFTDAHIEVDITYAEYFEMSVRLAEAMKRYGLNTNHRIVVCSENSLQFFMPVL<br>GALFIGVAVAPANDIYNERELLNSMGISQPTVVFVSKKGLQKILNVQKKLPIIQKIIIMDSKTDYQ<br>GFQSMYTFVTSHLPPGFNEYDFVPESFDRDKTIALIMNSSGSTGLPKGVALPHRTACVRFSHARDP<br>IFGNQIIPDTAILSVVPFHHGFGMFTTLGYLICGFRVVLMYRFEEELFLRSLQDYKIQSALLVPTL<br>FSFFAKSTLIDKYDLSNLHEIASGGAPLSKEVGEAVAKRFHLPGIRQGYGLTETTSAILITPEGDD<br>KPGAVGKVVPFFEAKVVDLDTGKTLGVNQRGELCVRGPMIMSGYVNNPEATNALIDKDGWLHSGDI<br>AYWDEDEHFFIVDRLKSLIKYKGYQVAPAELESILLQHPNIFDAGVAGLPDDDAGELPAAVVVLEH<br>GKTMTEKEIVDYVASQVTTAKKLRGGVVFVDEVPKGLTGKLDARKIREILIKAKKGGKIAVNS
	fLuc:Cul1(ΔC501)	pcDNA3.1-fluc:gs10:Cul1(dC501)
		EDAKNIKKGPAPFYPLEDGTAGEQLHKAMKRYALVPGTIAFTDAHIEVDITYAEYFEMSVRLAEAM<br>KRYGLNTNHRIVVCSENSLQFFMPVLGALFIGVAVAPANDIYNERELLNSMGISQPTVVFVSKKGL<br>QKILNVQKKLPIIQKIIIMDSKTDYQGFQSMYTFVTSHLPPGFNEYDFVPESFDRDKTIALIMNSS<br>GSTGLPKGVALPHRTACVRFSHARDPIFGNQIIPDTAILSVVPFHHGFGMFTTLGYLICGFRVVLM<br>YRFEEELFLRSLQDYKIQSALLVPTLFSFFAKSTLIDKYDLSNLHEIASGGAPLSKEVGEAVAKRF<br>HLPGIRQGYGLTETTSAILITPEGDDKPGAVGKVVPFFEAKVVDLDTGKTLGVNQRGELCVRGPMI<br>MSGYVNNPEATNALIDKDGWLHSGDIAYWDEDEHFFIVDRLKSLIKYKGYQVAPAELESILLQHPN<br>IFDAGVAGLPDDDAGELPAAVVVLEHGKTMTEKEIVDYVASQVTTAKKLRGGVVFVDEVPKGLTGK<br>LDARKIREILIKAKKGGKIAVNSGGSGGGSGGSSKLQRMFQDIGVSKDLNEQFKKHLTNSEPLDLD<br>FSIQVLSSGSWPFQQSCTFALPSELERSYQRFTAFYASRHSGRKLTWLYQLSKGELVTNCFKNRYT<br>LQASTFQMAILLQYNTEDAYTVQQLTDSTQIKMDILAQVLQILLKSKLLVLEDENANVDEVELKPD<br>TLIKLYLGYKNKKLRVNINVPMKTEQKQEQETTHKNIEEDRKLLIQAAIVRIMKMRKVLKHQQLLG<br>EVLTQLSSRFKPRVPVIKKCIDILIEKEYLERVDGEKDTYSYLA
	Rbx1:fLuc	pcDNA3.1-Rbx1:gs10:fluc
		AAAMDVDTPSGTNSGAGKKRFEVKKWNAVALWAWDIVVDNCAICRNHIMDLCIECQANQASATSEE<br>CTVAWGVCNHAFHFHCISRWLKTRQVCPLDNREWEFQKYGHGGSGGGSGGSEDAKNIKKGPAPFYP<br>LEDGTAGEQLHKAMKRYALVPGTIAFTDAHIEVDITYAEYFEMSVRLAEAMKRYGLNTNHRIVVCS<br>ENSLQFFMPVLGALFIGVAVAPANDIYNERELLNSMGISQPTVVFVSKKGLQKILNVQKKLPIIQK<br>IIIMDSKTDYQGFQSMYTFVTSHLPPGFNEYDFVPESFDRDKTIALIMNSSGSTGLPKGVALPHRT<br>ACVRFSHARDPIFGNQIIPDTAILSVVPFHHGFGMFTTLGYLICGFRVVLMYRFEEELFLRSLQDY<br>KIQSALLVPTLFSFFAKSTLIDKYDLSNLHEIASGGAPLSKEVGEAVAKRFHLPGIRQGYGLTETT<br>SAILITPEGDDKPGAVGKVVPFFEAKVVDLDTGKTLGVNQRGELCVRGPMIMSGYVNNPEATNALI<br>DKDGWLHSGDIAYWDEDEHFFIVDRLKSLIKYKGYQVAPAELESILLQHPNIFDAGVAGLPDDDAG

		ELPAAVVVLEHGKTMTEKEIVDYVASQVTTAKKLRGGVVFVDEVPKGLTGKLDARKIREILIKAKK<br>GGKIAVNS
	fLuc:CDC34	pcDNA3.1-fluc:gsg:CDC34
		EDAKNIKKGPAPFYPLEDGTAGEQLHKAMKRYALVPGTIAFTDAHIEVDITYAEYFEMSVRLAEAM<br>KRYGLNTNHRIVVCSENSLQFFMPVLGALFIGVAVAPANDIYNERELLNSMGISQPTVVFVSKKGL<br>QKILNVQKKLPIIQKIIIMDSKTDYQGFQSMYTFVTSHLPPGFNEYDFVPESFDRDKTIALIMNSS<br>GSTGLPKGVALPHRTACVRFSHARDPIFGNQIIPDTAILSVVPFHHGFGMFTTLGYLICGFRVVLM<br>YRFEEELFLRSLQDYKIQSALLVPTLFSFFAKSTLIDKYDLSNLHEIASGGAPLSKEVGEAVAKRF<br>HLPGIRQGYGLTETTSAILITPEGDDKPGAVGKVVPFFEAKVVDLDTGKTLGVNQRGELCVRGPMI<br>MSGYVNNPEATNALIDKDGWLHSGDIAYWDEDEHFFIVDRLKSLIKYKGYQVAPAELESILLQHPN<br>IFDAGVAGLPDDDAGELPAAVVVLEHGKTMTEKEIVDYVASQVTTAKKLRGGVVFVDEVPKGLTGK<br>LDARKIREILIKAKKGGKIAVNSGSGMARPLVPSSQKALLLELKGLQEEPVEGFRVTLVDEGDLYN<br>WEVAIFGPPNTYYEGGYFKARLKFPIDYPYSPPAFRFLTKMWHPNIYETGDVCISILHPPVDDPQS<br>GELPSERWNPTQNVRTILLSVISLLNEPNTFSPANVDASVMYRKWKESKGKDREYTDIIRKQVLGT<br>KVDAERDGVKVPTTLAEYCVKTKAPAPDEGSDLFYDDYYEDGEVEEEADSCFGDDEDDSGTEES
1d	Skp1(ΔC131):fLuc	pcDNA3.1-Skp1(ΔC131): gs10:fluc
		PSIKLQSSDGEIFEVDVEIAKQSVTIKTMLEDLGMDDEGDDDPVPLPNVNAAILKKVIQWCTHHKD<br>DPPPPEDDENKEKRTDDIPVWDQEFLKVDQGTLFELILAANYLDIKGLLDVTCKTVANMIKGKTGG<br>SGGGSGGSEDAKNIKKGPAPFYPLEDGTAGEQLHKAMKRYALVPGTIAFTDAHIEVDITYAEYFEM<br>SVRLAEAMKRYGLNTNHRIVVCSENSLQFFMPVLGALFIGVAVAPANDIYNERELLNSMGISQPTV<br>VFVSKKGLQKILNVQKKLPIIQKIIIMDSKTDYQGFQSMYTFVTSHLPPGFNEYDFVPESFDRDKT<br>IALIMNSSGSTGLPKGVALPHRTACVRFSHARDPIFGNQIIPDTAILSVVPFHHGFGMFTTLGYLI<br>CGFRVVLMYRFEEELFLRSLQDYKIQSALLVPTLFSFFAKSTLIDKYDLSNLHEIASGGAPLSKEV<br>GEAVAKRFHLPGIRQGYGLTETTSAILITPEGDDKPGAVGKVVPFFEAKVVDLDTGKTLGVNQRGE<br>LCVRGPMIMSGYVNNPEATNALIDKDGWLHSGDIAYWDEDEHFFIVDRLKSLIKYKGYQVAPAELE<br>SILLQHPNIFDAGVAGLPDDDAGELPAAVVVLEHGKTMTEKEIVDYVASQVTTAKKLRGGVVFVDE<br>VPKGLTGKLDARKIREILIKAKKGGKIAVNS
	Skp1(ΔC111):fLuc	pcDNA3.1-Skp1(dc111): gs10:fluc
		PSIKLQSSDGEIFEVDVEIAKQSVTIKTMLEDLGMDDEGDDDPVPLPNVNAAILKKVIQWCTHHKD<br>DPPPPEDDENKEKRTDDIPVWDQEFLKVDQGTLFELILAANYLDIGGSGGGSGGSEDAKNIKKGPA<br>PFYPLEDGTAGEQLHKAMKRYALVPGTIAFTDAHIEVDITYAEYFEMSVRLAEAMKRYGLNTNHRI<br>VVCSENSLQFFMPVLGALFIGVAVAPANDIYNERELLNSMGISQPTVVFVSKKGLQKILNVQKKLP<br>IIQKIIIMDSKTDYQGFQSMYTFVTSHLPPGFNEYDFVPESFDRDKTIALIMNSSGSTGLPKGVAL<br>PHRTACVRFSHARDPIFGNQIIPDTAILSVVPFHHGFGMFTTLGYLICGFRVVLMYRFEEELFLRS<br>LQDYKIQSALLVPTLFSFFAKSTLIDKYDLSNLHEIASGGAPLSKEVGEAVAKRFHLPGIRQGYGL<br>TETTSAILITPEGDDKPGAVGKVVPFFEAKVVDLDTGKTLGVNQRGELCVRGPMIMSGYVNNPEAT<br>NALIDKDGWLHSGDIAYWDEDEHFFIVDRLKSLIKYKGYQVAPAELESILLQHPNIFDAGVAGLPD<br>DDAGELPAAVVVLEHGKTMTEKEIVDYVASQVTTAKKLRGGVVFVDEVPKGLTGKLDARKIREILI<br>KAKKGGKIAVNS
	Skp1(ΔC94):fLuc	pcDNA3.1-Skp1(ΔC94): gs10:fluc
		PSIKLQSSDGEIFEVDVEIAKQSVTIKTMLEDLGMDDEGDDDPVPLPNVNAAILKKVIQWCTHHKD<br>DPPPPEDDENKEKRTDDIPVWDQEFLKGGSGGGSGGSEDAKNIKKGPAPFYPLEDGTAGEQLHKAM<br>KRYALVPGTIAFTDAHIEVDITYAEYFEMSVRLAEAMKRYGLNTNHRIVVCSENSLQFFMPVLGAL<br>FIGVAVAPANDIYNERELLNSMGISQPTVVFVSKKGLQKILNVQKKLPIIQKIIIMDSKTDYQGFQ<br>SMYTFVTSHLPPGFNEYDFVPESFDRDKTIALIMNSSGSTGLPKGVALPHRTACVRFSHARDPIFG<br>NQIIPDTAILSVVPFHHGFGMFTTLGYLICGFRVVLMYRFEEELFLRSLQDYKIQSALLVPTLFSF<br>FAKSTLIDKYDLSNLHEIASGGAPLSKEVGEAVAKRFHLPGIRQGYGLTETTSAILITPEGDDKPG<br>AVGKVVPFFEAKVVDLDTGKTLGVNQRGELCVRGPMIMSGYVNNPEATNALIDKDGWLHSGDIAYW<br>DEDEHFFIVDRLKSLIKYKGYQVAPAELESILLQHPNIFDAGVAGLPDDDAGELPAAVVVLEHGKT<br>MTEKEIVDYVASQVTTAKKLRGGVVFVDEVPKGLTGKLDARKIREILIKAKKGGKIAVNS
	Rbx1(ΔN36):fLuc	pcDNA3.1-Rbx1(ΔN36): gs10:fluc
		VVDNCAICRNHIMDLCIECQANQASATSEECTVAWGVCNHAFHFHCISRWLKTRQVCPLDNREWEF<br>QKYGHGGSGGGSGGSEDAKNIKKGPAPFYPLEDGTAGEQLHKAMKRYALVPGTIAFTDAHIEVDIT<br>YAEYFEMSVRLAEAMKRYGLNTNHRIVVCSENSLQFFMPVLGALFIGVAVAPANDIYNERELLNSM<br>GISQPTVVFVSKKGLQKILNVQKKLPIIQKIIIMDSKTDYQGFQSMYTFVTSHLPPGFNEYDFVPE<br>SFDRDKTIALIMNSSGSTGLPKGVALPHRTACVRFSHARDPIFGNQIIPDTAILSVVPFHHGFGMF<br>TTLGYLICGFRVVLMYRFEEELFLRSLQDYKIQSALLVPTLFSFFAKSTLIDKYDLSNLHEIASGG<br>APLSKEVGEAVAKRFHLPGIRQGYGLTETTSAILITPEGDDKPGAVGKVVPFFEAKVVDLDTGKTL<br>GVNQRGELCVRGPMIMSGYVNNPEATNALIDKDGWLHSGDIAYWDEDEHFFIVDRLKSLIKYKGYQ<br>VAPAELESILLQHPNIFDAGVAGLPDDDAGELPAAVVVLEHGKTMTEKEIVDYVASQVTTAKKLRG<br>GVVFVDEVPKGLTGKLDARKIREILIKAKKGGKIAVNS
2a	P3:fLuc	pcDNA3.1-P3: gs10:fluc
		SPEDEIQQLEEEIAQLEQKNAALKEKNQALKYGGGSGGGSGGSEDAKNIKKGPAPFYPLEDGTAGE<br>QLHKAMKRYALVPGTIAFTDAHIEVDITYAEYFEMSVRLAEAMKRYGLNTNHRIVVCSENSLQFFM<br>PVLGALFIGVAVAPANDIYNERELLNSMGISQPTVVFVSKKGLQKILNVQKKLPIIQKIIIMDSKT<br>DYQGFQSMYTFVTSHLPPGFNEYDFVPESFDRDKTIALIMNSSGSTGLPKGVALPHRTACVRFSHA<br>RDPIFGNQIIPDTAILSVVPFHHGFGMFTTLGYLICGFRVVLMYRFEEELFLRSLQDYKIQSALLV<br>PTLFSFFAKSTLIDKYDLSNLHEIASGGAPLSKEVGEAVAKRFHLPGIRQGYGLTETTSAILITPE<br>GDDKPGAVGKVVPFFEAKVVDLDTGKTLGVNQRGELCVRGPMIMSGYVNNPEATNALIDKDGWLHS<br>GDIAYWDEDEHFFIVDRLKSLIKYKGYQVAPAELESILLQHPNIFDAGVAGLPDDDAGELPAAVVV<br>LEHGKTMTEKEIVDYVASQVTTAKKLRGGVVFVDEVPKGLTGKLDARKIREILIKAKKGGKIAVNS
	F-box:P4	pcDNA3.1-F-box:gs10:P4

		RENFPGVSWDSLPDELLLGIFSCLCLPELLKVSGVCKRWYRLASDESLWQTLDLTGKNLHPDVTGR<br>LLSGGSGGGSGGSSPEDKIAQLKQKIQALKQENQQLEEENAALEYG
	Skp1:P4	pcDNA3.1-Skp1:gs10:P4<br>PSIKLQSSDGEIFEVDVEIAKQSVTIKTMLEDLGMDDEGDDDPVPLPNVNAAILKKVIQWCTHHKD<br>DPPPPEDDENKEKRTDDIPVWDQEFLKVDQGTLFELILAANYLDIKGLLDVTCKTVANMIKGKTPE<br>EIRKTFNIKNDFTEEEEAQVRKENQWCEEKGGSGGGSGGSSPEDKIAQLKQKIQALKQENQQLEEE<br>NAALEYG
	Skp1(ΔC131):P4	pcDNA3.1-Skp1(ΔC131):gs10:P4<br>PSIKLQSSDGEIFEVDVEIAKQSVTIKTMLEDLGMDDEGDDDPVPLPNVNAAILKKVIQWCTHHKD<br>DPPPPEDDENKEKRTDDIPVWDQEFLKVDQGTLFELILAANYLDIKGLLDVTCKTVANMIKGKTGG<br>SGGGSGGSSPEDKIAQLKQKIQALKQENQQLEEENAALEYG
	Skp1(ΔC111):P4	pcDNA3.1-Skp1(ΔC111):gs10:P4<br>PSIKLQSSDGEIFEVDVEIAKQSVTIKTMLEDLGMDDEGDDDPVPLPNVNAAILKKVIQWCTHHKD<br>DPPPPEDDENKEKRTDDIPVWDQEFLKVDQGTLFELILAANYLDIGGSGGGSGGSSPEDKIAQLKQ<br>KIQALKQENQQLEEENAALEYG
	Skp1(ΔC94):P4	pcDNA3.1-Skp1(ΔC94):gs10:P4<br>PSIKLQSSDGEIFEVDVEIAKQSVTIKTMLEDLGMDDEGDDDPVPLPNVNAAILKKVIQWCTHHKD<br>DPPPPEDDENKEKRTDDIPVWDQEFLKGGSGGGSGGSSPEDKIAQLKQKIQALKQENQQLEEENAA<br>LEYG
	P4:Cul1(ΔC501)	pcDNA3.1-P4:gs10:Cul1(ΔC501)<br>SPEDKIAQLKQKIQALKQENQQLEEENAALEYGGGSGGGSGGSSKLQRMFQDIGVSKDLNEQFKKH<br>LTNSEPLDLDFSIQVLSSGSWPFQQSCTFALPSELERSYQRFTAFYASRHSGRKLTWLYQLSKGEL<br>VTNCFKNRYTLQASTFQMAILLQYNTEDAYTVQQLTDSTQIKMDILAQVLQILLKSKLLVLEDENA<br>NVDEVELKPDTLIKLYLGYKNKKLRVNINVPMKTEQKQEQETTHKNIEEDRKLLIQAAIVRIMKMR<br>KVLKHQQLLGEVLTQLSSRFKPRVPVIKKCIDILIEKEYLERVDGEKDTYSYLA
	Rbx1:P4	pcDNA3.1-Rbx1:gs10:P4<br>AAAMDVDTPSGTNSGAGKKRFEVKKWNAVALWAWDIVVDNCAICRNHIMDLCIECQANQASATSEE<br>CTVAWGVCNHAFHFHCISRWLKTRQVCPLDNREWEFQKYGHGGSGGGSGGSSPEDKIAQLKQKIQA<br>LKQENQQLEEENAALEYG
	Rbx1(ΔN36):P4	Rbx1(ΔN36):gs10:P4<br>VVDNCAICRNHIMDLCIECQANQASATSEECTVAWGVCNHAFHFHCISRWLKTRQVCPLDNREWEF<br>QKYGHGGSGGGSGGSSPEDKIAQLKQKIQALKQENQQLEEENAALEYG
	P4:CDC34	pcDNA3.1-P4:gsg:CDC34<br>SPEDKIAQLKQKIQALKQENQQLEEENAALEYGGSGMARPLVPSSQKALLLELKGLQEEPVEGFRV<br>TLVDEGDLYNWEVAIFGPPNTYYEGGYFKARLKFPIDYPYSPPAFRFLTKMWHPNIYETGDVCISI<br>LHPPVDDPQSGELPSERWNPTQNVRTILLSVISLLNEPNTFSPANVDASVMYRKWKESKGKDREYT<br>DIIRKQVLGTKVDAERDGVKVPTTLAEYCVKTKAPAPDEGSDLFYDDYYEDGEVEEEADSCFGDDE<br>DDSGTEES
2b	P3:fLuc	Same as in Fig. 2a
	P5:fLuc	pcDNA3.1-P5: gs10:fluc<br>SPEDENAALEEKIAQLKQKNAALKEEIQALEYGGGSGGGSGGSEDAKNIKKGPAPFYPLEDGTAGE<br>QLHKAMKRYALVPGTIAFTDAHIEVDITYAEYFEMSVRLAEAMKRYGLNTNHRIVVCSENSLQFFM<br>PVLGALFIGVAVAPANDIYNERELLNSMGISQPTVVFVSKKGLQKILNVQKKLPIIQKIIIMDSKT<br>DYQGFQSMYTFVTSHLPPGFNEYDFVPESFDRDKTIALIMNSSGSTGLPKGVALPHRTACVRFSHA<br>RDPIFGNQIIPDTAILSVVPFHHGFGMFTTLGYLICGFRVVLMYRFEEELFLRSLQDYKIQSALLV<br>PTLFSFFAKSTLIDKYDLSNLHEIASGGAPLSKEVGEAVAKRFHLPGIRQGYGLTETTSAILITPE<br>GDDKPGAVGKVVPFFEAKVVDLDTGKTLGVNQRGELCVRGPMIMSGYVNNPEATNALIDKDGWLHS<br>GDIAYWDEDEHFFIVDRLKSLIKYKGYQVAPAELESILLQHPNIFDAGVAGLPDDDAGELPAAVVV<br>LEHGKTMTEKEIVDYVASQVTTAKKLRGGVVFVDEVPKGLTGKLDARKIREILIKAKKGGKIAVNS
	P7:fLuc	pcDNA3.1-P7: gs10:fluc<br>SPEDEIQALEEKNAQLKQEIAALEEKNQALKYGGGSGGGSGGSEDAKNIKKGPAPFYPLEDGTAGE<br>QLHKAMKRYALVPGTIAFTDAHIEVDITYAEYFEMSVRLAEAMKRYGLNTNHRIVVCSENSLQFFM<br>PVLGALFIGVAVAPANDIYNERELLNSMGISQPTVVFVSKKGLQKILNVQKKLPIIQKIIIMDSKT<br>DYQGFQSMYTFVTSHLPPGFNEYDFVPESFDRDKTIALIMNSSGSTGLPKGVALPHRTACVRFSHA<br>RDPIFGNQIIPDTAILSVVPFHHGFGMFTTLGYLICGFRVVLMYRFEEELFLRSLQDYKIQSALLV<br>PTLFSFFAKSTLIDKYDLSNLHEIASGGAPLSKEVGEAVAKRFHLPGIRQGYGLTETTSAILITPE<br>GDDKPGAVGKVVPFFEAKVVDLDTGKTLGVNQRGELCVRGPMIMSGYVNNPEATNALIDKDGWLHS<br>GDIAYWDEDEHFFIVDRLKSLIKYKGYQVAPAELESILLQHPNIFDAGVAGLPDDDAGELPAAVVV<br>LEHGKTMTEKEIVDYVASQVTTAKKLRGGVVFVDEVPKGLTGKLDARKIREILIKAKKGGKIAVNS
	F-box:P4	Same as in Fig. 2a
	F-box:P6	pcDNA3.1-F-box:gs10:P6<br>RENFPGVSWDSLPDELLLGIFSCLCLPELLKVSGVCKRWYRLASDESLWQTLDLTGKNLHPDVTGR<br>LLSGGSGGGSGGSSPEDKNAALKEEIQALEEENQALEEKIAQLKYG
	F-box:P8	pcDNA3.1-F-box:gs10:P8<br>RENFPGVSWDSLPDELLLGIFSCLCLPELLKVSGVCKRWYRLASDESLWQTLDLTGKNLHPDVTGR<br>LLSGGSGGGSGGSSPEDKIAQLKEENQQLEQKIQALKEENAALEYG
	Skp1(ΔC111):P4	Same as in Fig. 2a

	Skp1(ΔC111):P6	pcDNA3.1-Skp1(ΔC111):gs10:P6<br>PSIKLQSSDGEIFEVDVEIAKQSVTIKTMLEDLGMDDEGDDDPVPLPNVNAAILKKVIQWCTHHKD<br>DPPPPEDDENKEKRTDDIPVWDQEFLKVDQGTLFELILAANYLDIGGSGGGSGGSSPEDKNAALKE<br>EIQALEEENQALEEKIAQLKYG
	Skp1(ΔC111):P8	pcDNA3.1-Skp1(ΔC111):gs10:P8<br>PSIKLQSSDGEIFEVDVEIAKQSVTIKTMLEDLGMDDEGDDDPVPLPNVNAAILKKVIQWCTHHKD<br>DPPPPEDDENKEKRTDDIPVWDQEFLKVDQGTLFELILAANYLDIGGSGGGSGGSSPEDKIAQLKE<br>ENQQLEQKIQALKEENAALEYG
	P4:Cul1(ΔC501)	Same as in Fig. 2a
	P6:Cul1(ΔC501)	pcDNA3.1-P6: gs10:Cul1(ΔC501)<br>SPEDKNAALKEEIQALEEENQALEEKIAQLKYGGGSGGGSGGSSKLQRMFQDIGVSKDLNEQFKKH<br>LTNSEPLDLDFSIQVLSSGSWPFQQSCTFALPSELERSYQRFTAFYASRHSGRKLTWLYQLSKGEL<br>VTNCFKNRYTLQASTFQMAILLQYNTEDAYTVQQLTDSTQIKMDILAQVLQILLKSKLLVLEDENA<br>NVDEVELKPDTLIKLYLGYKNKKLRVNINVPMKTEQKQEQETTHKNIEEDRKLLIQAAIVRIMKMR<br>KVLKHQQLLGEVLTQLSSRFKPRVPVIKKCIDILIEKEYLERVDGEKDTYSYLA
	P8:Cul1(ΔC501)	pcDNA3.1-P8: gs10:Cul1(ΔC501)<br>SPEDKIAQLKEENQQLEQKIQALKEENAALEYGGGSGGGSGGSSKLQRMFQDIGVSKDLNEQFKKH<br>LTNSEPLDLDFSIQVLSSGSWPFQQSCTFALPSELERSYQRFTAFYASRHSGRKLTWLYQLSKGEL<br>VTNCFKNRYTLQASTFQMAILLQYNTEDAYTVQQLTDSTQIKMDILAQVLQILLKSKLLVLEDENA<br>NVDEVELKPDTLIKLYLGYKNKKLRVNINVPMKTEQKQEQETTHKNIEEDRKLLIQAAIVRIMKMR<br>KVLKHQQLLGEVLTQLSSRFKPRVPVIKKCIDILIEKEYLERVDGEKDTYSYLA
	Rbx1:P4	Same as in Fig. 2a
	Rbx1:P6	pcDNA3.1-Rbx1:gs10:P6<br>AAAMDVDTPSGTNSGAGKKRFEVKKWNAVALWAWDIVVDNCAICRNHIMDLCIECQANQASATSEE<br>CTVAWGVCNHAFHFHCISRWLKTRQVCPLDNREWEFQKYGHGGSGGGSGGSSPEDKNAALKEEIQA<br>LEEENQALEEKIAQLKYG
	Rbx1:P8	pcDNA3.1-Rbx1:gs10:P8<br>AAAMDVDTPSGTNSGAGKKRFEVKKWNAVALWAWDIVVDNCAICRNHIMDLCIECQANQASATSEE<br>CTVAWGVCNHAFHFHCISRWLKTRQVCPLDNREWEFQKYGHGGSGGGSGGSSPEDKIAQLKEENQQ<br>LEQKIQALKEENAALEYG
	P4:CDC34	Same as in Fig. 2a
	P6:CDC34	pcDNA3.1-P6: gsg:CDC34<br>SPEDKNAALKEEIQALEEENQALEEKIAQLKYGGSGMARPLVPSSQKALLLELKGLQEEPVEGFRV<br>TLVDEGDLYNWEVAIFGPPNTYYEGGYFKARLKFPIDYPYSPPAFRFLTKMWHPNIYETGDVCISI<br>LHPPVDDPQSGELPSERWNPTQNVRTILLSVISLLNEPNTFSPANVDASVMYRKWKESKGKDREYT<br>DIIRKQVLGTKVDAERDGVKVPTTLAEYCVKTKAPAPDEGSDLFYDDYYEDGEVEEEADSCFGDDE<br>DDSGTEES
	P8:CDC34	pcDNA3.1-P8: gsg:CDC34<br>SPEDKIAQLKEENQQLEQKIQALKEENAALEYGGSGMARPLVPSSQKALLLELKGLQEEPVEGFRV<br>TLVDEGDLYNWEVAIFGPPNTYYEGGYFKARLKFPIDYPYSPPAFRFLTKMWHPNIYETGDVCISI<br>LHPPVDDPQSGELPSERWNPTQNVRTILLSVISLLNEPNTFSPANVDASVMYRKWKESKGKDREYT<br>DIIRKQVLGTKVDAERDGVKVPTTLAEYCVKTKAPAPDEGSDLFYDDYYEDGEVEEEADSCFGDDE<br>DDSGTEES
2c	P3:gsg:fLuc	pcDNA3.1-P3:gsg:fLuc<br>SPEDEIQQLEEEIAQLEQKNAALKEKNQALKYGGSGEDAKNIKKGPAPFYPLEDGTAGEQLHKAMK<br>RYALVPGTIAFTDAHIEVDITYAEYFEMSVRLAEAMKRYGLNTNHRIVVCSENSLQFFMPVLGALF<br>IGVAVAPANDIYNERELLNSMGISQPTVVFVSKKGLQKILNVQKKLPIIQKIIIMDSKTDYQGFQS<br>MYTFVTSHLPPGFNEYDFVPESFDRDKTIALIMNSSGSTGLPKGVALPHRTACVRFSHARDPIFGN<br>QIIPDTAILSVVPFHHGFGMFTTLGYLICGFRVVLMYRFEEELFLRSLQDYKIQSALLVPTLFSFF<br>AKSTLIDKYDLSNLHEIASGGAPLSKEVGEAVAKRFHLPGIRQGYGLTETTSAILITPEGDDKPGA<br>VGKVVPFFEAKVVDLDTGKTLGVNQRGELCVRGPMIMSGYVNNPEATNALIDKDGWLHSGDIAYWD<br>EDEHFFIVDRLKSLIKYKGYQVAPAELESILLQHPNIFDAGVAGLPDDDAGELPAAVVVLEHGKTM<br>TEKEIVDYVASQVTTAKKLRGGVVFVDEVPKGLTGKLDARKIREILIKAKKGGKIAVNS
	P3:gs10:fLuc	Same as in Fig. 2a
	P3:gs30:fLuc	pcDNA3.1-P3:gs30:fLuc<br>SPEDEIQQLEEEIAQLEQKNAALKEKNQALKYGGGSGGGSGGSGGSGGGSGGSGGSGGGSGGSEDA<br>KNIKKGPAPFYPLEDGTAGEQLHKAMKRYALVPGTIAFTDAHIEVDITYAEYFEMSVRLAEAMKRY<br>GLNTNHRIVVCSENSLQFFMPVLGALFIGVAVAPANDIYNERELLNSMGISQPTVVFVSKKGLQKI<br>LNVQKKLPIIQKIIIMDSKTDYQGFQSMYTFVTSHLPPGFNEYDFVPESFDRDKTIALIMNSSGST<br>GLPKGVALPHRTACVRFSHARDPIFGNQIIPDTAILSVVPFHHGFGMFTTLGYLICGFRVVLMYRF<br>EEELFLRSLQDYKIQSALLVPTLFSFFAKSTLIDKYDLSNLHEIASGGAPLSKEVGEAVAKRFHLP<br>GIRQGYGLTETTSAILITPEGDDKPGAVGKVVPFFEAKVVDLDTGKTLGVNQRGELCVRGPMIMSG<br>YVNNPEATNALIDKDGWLHSGDIAYWDEDEHFFIVDRLKSLIKYKGYQVAPAELESILLQHPNIFD<br>AGVAGLPDDDAGELPAAVVVLEHGKTMTEKEIVDYVASQVTTAKKLRGGVVFVDEVPKGLTGKLDA<br>RKIREILIKAKKGGKIAVNS
	F-box:gsg:P4	pcDNA3.1-F-box:gsg:P4<br>RENFPGVSWDSLPDELLLGIFSCLCLPELLKVSGVCKRWYRLASDESLWQTLDLTGKNLHPDVTGR<br>LLSGSGSPEDKIAQLKQKIQALKQENQQLEEENAALEYG

	F-box:gs10:P4	Same as in Fig. 2a
	F-box:gs30:P4	pcDNA3.1-F-box:gs30:P4<br>RENFPGVSWDSLPDELLLGIFSCLCLPELLKVSGVCKRWYRLASDESLWQTLDLTGKNLHPDVTGR<br>LLSGGSGGGSGGSGGSGGGSGGSGGSGGGSGGSSPEDKIAQLKQKIQALKQENQQLEEENAALEYG
	Skp1(ΔC111):gsg:P4	pcDNA3.1-Skp1(ΔC111):gsg:P4<br>PSIKLQSSDGEIFEVDVEIAKQSVTIKTMLEDLGMDDEGDDDPVPLPNVNAAILKKVIQWCTHHKD<br>DPPPPEDDENKEKRTDDIPVWDQEFLKVDQGTLFELILAANYLDIGSGSPEDKIAQLKQKIQALKQ<br>ENQQLEEENAALEYG
	Skp1(ΔC111):gs10:P4	Same as in Fig. 2a
	Skp1(ΔC111):gs30:P4	pcDNA3.1-Skp1(ΔC111):gs30:P4<br>PSIKLQSSDGEIFEVDVEIAKQSVTIKTMLEDLGMDDEGDDDPVPLPNVNAAILKKVIQWCTHHKD<br>DPPPPEDDENKEKRTDDIPVWDQEFLKVDQGTLFELILAANYLDIGGSGGGSGGSGGSGGGSGGSG<br>GSGGGSGGSSPEDKIAQLKQKIQALKQENQQLEEENAALEYG
	P4:gsg:Cul1(ΔC501)	pcDNA3.1-P4: gsg:Cul1(ΔC501)<br>SPEDKIAQLKQKIQALKQENQQLEEENAALEYGGSGSKLQRMFQDIGVSKDLNEQFKKHLTNSEPL<br>DLDFSIQVLSSGSWPFQQSCTFALPSELERSYQRFTAFYASRHSGRKLTWLYQLSKGELVTNCFKN<br>RYTLQASTFQMAILLQYNTEDAYTVQQLTDSTQIKMDILAQVLQILLKSKLLVLEDENANVDEVEL<br>KPDTLIKLYLGYKNKKLRVNINVPMKTEQKQEQETTHKNIEEDRKLLIQAAIVRIMKMRKVLKHQQ<br>LLGEVLTQLSSRFKPRVPVIKKCIDILIEKEYLERVDGEKDTYSYLA
	P4:gs10:Cul1(ΔC501)	Same as in Fig. 2a
	P4:gs30:Cul1(ΔC501)	pcDNA3.1-P4: gs30:Cul1(ΔC501)<br>SPEDKIAQLKQKIQALKQENQQLEEENAALEYGGGSGGGSGGSGGSGGGSGGSGGSGGGSGGSSKL<br>QRMFQDIGVSKDLNEQFKKHLTNSEPLDLDFSIQVLSSGSWPFQQSCTFALPSELERSYQRFTAFY<br>ASRHSGRKLTWLYQLSKGELVTNCFKNRYTLQASTFQMAILLQYNTEDAYTVQQLTDSTQIKMDIL<br>AQVLQILLKSKLLVLEDENANVDEVELKPDTLIKLYLGYKNKKLRVNINVPMKTEQKQEQETTHKN<br>IEEDRKLLIQAAIVRIMKMRKVLKHQQLLGEVLTQLSSRFKPRVPVIKKCIDILIEKEYLERVDGE<br>KDTYSYLA
	Rbx1:gsg:P4	pcDNA3.1-Rbx1: gsg:P4<br>AAAMDVDTPSGTNSGAGKKRFEVKKWNAVALWAWDIVVDNCAICRNHIMDLCIECQANQASATSEE<br>CTVAWGVCNHAFHFHCISRWLKTRQVCPLDNREWEFQKYGHGSGSPEDKIAQLKQKIQALKQENQQ<br>LEEENAALEYG
	Rbx1:gs10:P4	Same as in Fig. 2a
	Rbx1:gs30:P4	pcDNA3.1-Rbx1: gs30:P4<br>AAAMDVDTPSGTNSGAGKKRFEVKKWNAVALWAWDIVVDNCAICRNHIMDLCIECQANQASATSEE<br>CTVAWGVCNHAFHFHCISRWLKTRQVCPLDNREWEFQKYGHGGSGGGSGGSGGSGGGSGGSGGSGG<br>GSGGSSPEDKIAQLKQKIQALKQENQQLEEENAALEYG
	P4:gsg:CDC34	pcDNA3.1-P4: gsg:CDC34<br>SPEDKIAQLKQKIQALKQENQQLEEENAALEYGGSGMARPLVPSSQKALLLELKGLQEEPVEGFRV<br>TLVDEGDLYNWEVAIFGPPNTYYEGGYFKARLKFPIDYPYSPPAFRFLTKMWHPNIYETGDVCISI<br>LHPPVDDPQSGELPSERWNPTQNVRTILLSVISLLNEPNTFSPANVDASVMYRKWKESKGKDREYT<br>DIIRKQVLGTKVDAERDGVKVPTTLAEYCVKTKAPAPDEGSDLFYDDYYEDGEVEEEADSCFGDDE<br>DDSGTEES
	P4:gs10:CDC34	Same as in Fig. 2a
	P4:gs30:CDC34	pcDNA3.1-P4: gs30:CDC34<br>SPEDKIAQLKQKIQALKQENQQLEEENAALEYGGGSGGGSGGSGGSGGGSGGSGGSGGGSGGSMAR<br>PLVPSSQKALLLELKGLQEEPVEGFRVTLVDEGDLYNWEVAIFGPPNTYYEGGYFKARLKFPIDYP<br>YSPPAFRFLTKMWHPNIYETGDVCISILHPPVDDPQSGELPSERWNPTQNVRTILLSVISLLNEPN<br>TFSPANVDASVMYRKWKESKGKDREYTDIIRKQVLGTKVDAERDGVKVPTTLAEYCVKTKAPAPDE<br>GSDLFYDDYYEDGEVEEEADSCFGDDEDDSGTEES
3a	AP4:TEVs:P3:fLuc	pcDNA3.1-AP4:gs6:TEVs:gs6:P3:gs10:fluc<br>SPEDELAANEEELQQNEQKLAQIKQKLQAIKYGGGSGGSENLYFQSGGSGGSSPEDEIQQLEEEIA<br>QLEQKNAALKEKNQALKYGGGSGGGSGGSEDAKNIKKGPAPFYPLEDGTAGEQLHKAMKRYALVPG<br>TIAFTDAHIEVDITYAEYFEMSVRLAEAMKRYGLNTNHRIVVCSENSLQFFMPVLGALFIGVAVAP<br>ANDIYNERELLNSMGISQPTVVFVSKKGLQKILNVQKKLPIIQKIIIMDSKTDYQGFQSMYTFVTS<br>HLPPGFNEYDFVPESFDRDKTIALIMNSSGSTGLPKGVALPHRTACVRFSHARDPIFGNQIIPDTA<br>ILSVVPFHHGFGMFTTLGYLICGFRVVLMYRFEEELFLRSLQDYKIQSALLVPTLFSFFAKSTLID<br>KYDLSNLHEIASGGAPLSKEVGEAVAKRFHLPGIRQGYGLTETTSAILITPEGDDKPGAVGKVVPF<br>FEAKVVDLDTGKTLGVNQRGELCVRGPMIMSGYVNNPEATNALIDKDGWLHSGDIAYWDEDEHFFI<br>VDRLKSLIKYKGYQVAPAELESILLQHPNIFDAGVAGLPDDDAGELPAAVVVLEHGKTMTEKEIVD<br>YVASQVTTAKKLRGGVVFVDEVPKGLTGKLDARKIREILIKAKKGGKIAVNS
	F-box:P4	Same as in Fig. 2a
	Skp1(ΔC111):P4	Same as in Fig. 2a
	P4:Cul1(ΔC501)	Same as in Fig. 2a
	Rbx1:P4	Same as in Fig. 2a
	P4:CDC34	Same as in Fig. 2a

	TEVp	pcDNA3.1-TEVp<br>GESLFKGPRDYNPISSTICHLTNESDGHTTSLYGIGFGPFIITNKHLFRRNNGTLLVQSLHGVFKV<br>KNTTTLQQHLIDGRDMIIIRMPKDFPPFPQKLKFREPQREERICLVTTNFQTKSMSSMVSDTSCTF<br>PSSDGIFWKHWIQTKDGQCGSPLVSTRDGFIVGIHSASNFTNTNNYFTSVPKNFMELLTNQEAQQW<br>VSGWRLNADSVLWGGHKVFMSKPEEPFQPVKEATQLMSELVYSQYPYDVPDYA
3b	F-box:TEVs:fLuc	pcDNA3.1-f-box:gs6:TEVs:gs6:fluc<br>RENFPGVSWDSLPDELLLGIFSCLCLPELLKVSGVCKRWYRLASDESLWQTLDLTGKNLHPDVTGR<br>LLSGGSGGSENLYFQSGGSGGSEDAKNIKKGPAPFYPLEDGTAGEQLHKAMKRYALVPGTIAFTDA<br>HIEVDITYAEYFEMSVRLAEAMKRYGLNTNHRIVVCSENSLQFFMPVLGALFIGVAVAPANDIYNE<br>RELLNSMGISQPTVVFVSKKGLQKILNVQKKLPIIQKIIIMDSKTDYQGFQSMYTFVTSHLPPGFN<br>EYDFVPESFDRDKTIALIMNSSGSTGLPKGVALPHRTACVRFSHARDPIFGNQIIPDTAILSVVPF<br>HHGFGMFTTLGYLICGFRVVLMYRFEEELFLRSLQDYKIQSALLVPTLFSFFAKSTLIDKYDLSNL<br>HEIASGGAPLSKEVGEAVAKRFHLPGIRQGYGLTETTSAILITPEGDDKPGAVGKVVPFFEAKVVD<br>LDTGKTLGVNQRGELCVRGPMIMSGYVNNPEATNALIDKDGWLHSGDIAYWDEDEHFFIVDRLKSL<br>IKYKGYQVAPAELESILLQHPNIFDAGVAGLPDDDAGELPAAVVVLEHGKTMTEKEIVDYVASQVT<br>TAKKLRGGVVFVDEVPKGLTGKLDARKIREILIKAKKGGKIAVNS
	Skp1(ΔC111):TEVs:fLcuc	pcDNA3.1-skp1(dc111): gs6:TEVs:gs6:fluc<br>PSIKLQSSDGEIFEVDVEIAKQSVTIKTMLEDLGMDDEGDDDPVPLPNVNAAILKKVIQWCTHHKD<br>DPPPPEDDENKEKRTDDIPVWDQEFLKVDQGTLFELILAANYLDIGGSGGSENLYFQSGGSGGSED<br>AKNIKKGPAPFYPLEDGTAGEQLHKAMKRYALVPGTIAFTDAHIEVDITYAEYFEMSVRLAEAMKR<br>YGLNTNHRIVVCSENSLQFFMPVLGALFIGVAVAPANDIYNERELLNSMGISQPTVVFVSKKGLQK<br>ILNVQKKLPIIQKIIIMDSKTDYQGFQSMYTFVTSHLPPGFNEYDFVPESFDRDKTIALIMNSSGS<br>TGLPKGVALPHRTACVRFSHARDPIFGNQIIPDTAILSVVPFHHGFGMFTTLGYLICGFRVVLMYR<br>FEEELFLRSLQDYKIQSALLVPTLFSFFAKSTLIDKYDLSNLHEIASGGAPLSKEVGEAVAKRFHL<br>PGIRQGYGLTETTSAILITPEGDDKPGAVGKVVPFFEAKVVDLDTGKTLGVNQRGELCVRGPMIMS<br>GYVNNPEATNALIDKDGWLHSGDIAYWDEDEHFFIVDRLKSLIKYKGYQVAPAELESILLQHPNIF<br>DAGVAGLPDDDAGELPAAVVVLEHGKTMTEKEIVDYVASQVTTAKKLRGGVVFVDEVPKGLTGKLD<br>ARKIREILIKAKKGGKIAVNS
	fLuc:TEVs:Cul1(ΔC501)	pcDNA3.1-fluc:gs6:TEVs:gs6:Cul1(dC501)<br>EDAKNIKKGPAPFYPLEDGTAGEQLHKAMKRYALVPGTIAFTDAHIEVDITYAEYFEMSVRLAEAM<br>KRYGLNTNHRIVVCSENSLQFFMPVLGALFIGVAVAPANDIYNERELLNSMGISQPTVVFVSKKGL<br>QKILNVQKKLPIIQKIIIMDSKTDYQGFQSMYTFVTSHLPPGFNEYDFVPESFDRDKTIALIMNSS<br>GSTGLPKGVALPHRTACVRFSHARDPIFGNQIIPDTAILSVVPFHHGFGMFTTLGYLICGFRVVLM<br>YRFEEELFLRSLQDYKIQSALLVPTLFSFFAKSTLIDKYDLSNLHEIASGGAPLSKEVGEAVAKRF<br>HLPGIRQGYGLTETTSAILITPEGDDKPGAVGKVVPFFEAKVVDLDTGKTLGVNQRGELCVRGPMI<br>MSGYVNNPEATNALIDKDGWLHSGDIAYWDEDEHFFIVDRLKSLIKYKGYQVAPAELESILLQHPN<br>IFDAGVAGLPDDDAGELPAAVVVLEHGKTMTEKEIVDYVASQVTTAKKLRGGVVFVDEVPKGLTGK<br>LDARKIREILIKAKKGGKIAVNSGGSGGSENLYFQSGGSGGSSKLQRMFQDIGVSKDLNEQFKKHL<br>TNSEPLDLDFSIQVLSSGSWPFQQSCTFALPSELERSYQRFTAFYASRHSGRKLTWLYQLSKGELV<br>TNCFKNRYTLQASTFQMAILLQYNTEDAYTVQQLTDSTQIKMDILAQVLQILLKSKLLVLEDENAN<br>VDEVELKPDTLIKLYLGYKNKKLRVNINVPMKTEQKQEQETTHKNIEEDRKLLIQAAIVRIMKMRK<br>VLKHQQLLGEVLTQLSSRFKPRVPVIKKCIDILIEKEYLERVDGEKDTYSYLA
	Rbx1:TEVs:fLuc	pcDNA3.1-rbx1: gs6:TEVs:gs6:fluc<br>AAAMDVDTPSGTNSGAGKKRFEVKKWNAVALWAWDIVVDNCAICRNHIMDLCIECQANQASATSEE<br>CTVAWGVCNHAFHFHCISRWLKTRQVCPLDNREWEFQKYGHGGSGGSENLYFQSGGSGGSEDAKNI<br>KKGPAPFYPLEDGTAGEQLHKAMKRYALVPGTIAFTDAHIEVDITYAEYFEMSVRLAEAMKRYGLN<br>TNHRIVVCSENSLQFFMPVLGALFIGVAVAPANDIYNERELLNSMGISQPTVVFVSKKGLQKILNV<br>QKKLPIIQKIIIMDSKTDYQGFQSMYTFVTSHLPPGFNEYDFVPESFDRDKTIALIMNSSGSTGLP<br>KGVALPHRTACVRFSHARDPIFGNQIIPDTAILSVVPFHHGFGMFTTLGYLICGFRVVLMYRFEEE<br>LFLRSLQDYKIQSALLVPTLFSFFAKSTLIDKYDLSNLHEIASGGAPLSKEVGEAVAKRFHLPGIR<br>QGYGLTETTSAILITPEGDDKPGAVGKVVPFFEAKVVDLDTGKTLGVNQRGELCVRGPMIMSGYVN<br>NPEATNALIDKDGWLHSGDIAYWDEDEHFFIVDRLKSLIKYKGYQVAPAELESILLQHPNIFDAGV<br>AGLPDDDAGELPAAVVVLEHGKTMTEKEIVDYVASQVTTAKKLRGGVVFVDEVPKGLTGKLDARKI<br>REILIKAKKGGKIAVNS
	fLuc:TEVs:CDC34	pcDNA3.1-fluc:gs6:TEVs:gs6:CDC34<br>EDAKNIKKGPAPFYPLEDGTAGEQLHKAMKRYALVPGTIAFTDAHIEVDITYAEYFEMSVRLAEAM<br>KRYGLNTNHRIVVCSENSLQFFMPVLGALFIGVAVAPANDIYNERELLNSMGISQPTVVFVSKKGL<br>QKILNVQKKLPIIQKIIIMDSKTDYQGFQSMYTFVTSHLPPGFNEYDFVPESFDRDKTIALIMNSS<br>GSTGLPKGVALPHRTACVRFSHARDPIFGNQIIPDTAILSVVPFHHGFGMFTTLGYLICGFRVVLM<br>YRFEEELFLRSLQDYKIQSALLVPTLFSFFAKSTLIDKYDLSNLHEIASGGAPLSKEVGEAVAKRF<br>HLPGIRQGYGLTETTSAILITPEGDDKPGAVGKVVPFFEAKVVDLDTGKTLGVNQRGELCVRGPMI<br>MSGYVNNPEATNALIDKDGWLHSGDIAYWDEDEHFFIVDRLKSLIKYKGYQVAPAELESILLQHPN<br>IFDAGVAGLPDDDAGELPAAVVVLEHGKTMTEKEIVDYVASQVTTAKKLRGGVVFVDEVPKGLTGK<br>LDARKIREILIKAKKGGKIAVNSGGSGGSENLYFQSGGSGGSMARPLVPSSQKALLLELKGLQEEP<br>VEGFRVTLVDEGDLYNWEVAIFGPPNTYYEGGYFKARLKFPIDYPYSPPAFRFLTKMWHPNIYETG<br>DVCISILHPPVDDPQSGELPSERWNPTQNVRTILLSVISLLNEPNTFSPANVDASVMYRKWKESKG<br>KDREYTDIIRKQVLGTKVDAERDGVKVPTTLAEYCVKTKAPAPDEGSDLFYDDYYEDGEVEEEADS<br>CFGDDEDDSGTEES
3c	FKBP:fLuc	pcDNA3.1-FKBP:gs10:fLuc<br>GVQVETISPGDGRTFPKRGQTCVVHYTGMLEDGKKFDSSRDRNKPFKFMLGKQEVIRGWEEGVAQM<br>SVGQRAKLTISPDYAYGATGHPGIIPPHATLVFDVELLKLEGGSGGGSGGSEDAKNIKKGPAPFYP<br>LEDGTAGEQLHKAMKRYALVPGTIAFTDAHIEVDITYAEYFEMSVRLAEAMKRYGLNTNHRIVVCS

		ENSLQFFMPVLGALFIGVAVAPANDIYNERELLNSMGISQPTVVFVSKKGLQKILNVQKKLPIIQK<br>IIIMDSKTDYQGFQSMYTFVTSHLPPGFNEYDFVPESFDRDKTIALIMNSSGSTGLPKGVALPHRT<br>ACVRFSHARDPIFGNQIIPDTAILSVVPFHHGFGMFTTLGYLICGFRVVLMYRFEEELFLRSLQDY<br>KIQSALLVPTLFSFFAKSTLIDKYDLSNLHEIASGGAPLSKEVGEAVAKRFHLPGIRQGYGLTETT<br>SAILITPEGDDKPGAVGKVVPFFEAKVVDLDTGKTLGVNQRGELCVRGPMIMSGYVNNPEATNALI<br>DKDGWLHSGDIAYWDEDEHFFIVDRLKSLIKYKGYQVAPAELESILLQHPNIFDAGVAGLPDDDAG<br>ELPAAVVVLEHGKTMTEKEIVDYVASQVTTAKKLRGGVVFVDEVPKGLTGKLDARKIREILIKAKK<br>GGKIAVNS
	fLuc:FKBP	pcDNA3.1-fLuc:gs10:FKBP
		EDAKNIKKGPAPFYPLEDGTAGEQLHKAMKRYALVPGTIAFTDAHIEVDITYAEYFEMSVRLAEAM<br>KRYGLNTNHRIVVCSENSLQFFMPVLGALFIGVAVAPANDIYNERELLNSMGISQPTVVFVSKKGL<br>QKILNVQKKLPIIQKIIIMDSKTDYQGFQSMYTFVTSHLPPGFNEYDFVPESFDRDKTIALIMNSS<br>GSTGLPKGVALPHRTACVRFSHARDPIFGNQIIPDTAILSVVPFHHGFGMFTTLGYLICGFRVVLM<br>YRFEEELFLRSLQDYKIQSALLVPTLFSFFAKSTLIDKYDLSNLHEIASGGAPLSKEVGEAVAKRF<br>HLPGIRQGYGLTETTSAILITPEGDDKPGAVGKVVPFFEAKVVDLDTGKTLGVNQRGELCVRGPMI<br>MSGYVNNPEATNALIDKDGWLHSGDIAYWDEDEHFFIVDRLKSLIKYKGYQVAPAELESILLQHPN<br>IFDAGVAGLPDDDAGELPAAVVVLEHGKTMTEKEIVDYVASQVTTAKKLRGGVVFVDEVPKGLTGK<br>LDARKIREILIKAKKGGKIAVNSGGSGGGSGGSGVQVETISPGDGRTFPKRGQTCVVHYTGMLEDG<br>KKFDSSRDRNKPFKFMLGKQEVIRGWEEGVAQMSVGQRAKLTISPDYAYGATGHPGIIPPHATLVF<br>DVELLKLE
	F-box:FRB	pcDNA3.1-F-box:gs10:FRB
		RENFPGVSWDSLPDELLLGIFSCLCLPELLKVSGVCKRWYRLASDESLWQTLDLTGKNLHPDVTGR<br>LLSGGSGGGSGGSASRILWHEMWHEGLEEASRLYFGERNVKGMFEVLEPLHAMMERGPQTLKETSF<br>NQAYGRDLMEAQEWCRKYMKSGNVKDLLQAWDLYYHVFRRISK
	Skp1(ΔC111):FRB	pcDNA3.1-Skp1(ΔC111): gs10:FRB
		PSIKLQSSDGEIFEVDVEIAKQSVTIKTMLEDLGMDDEGDDDPVPLPNVNAAILKKVIQWCTHHKD<br>DPPPPEDDENKEKRTDDIPVWDQEFLKVDQGTLFELILAANYLDIGGSGGGSGGSASRILWHEMWH<br>EGLEEASRLYFGERNVKGMFEVLEPLHAMMERGPQTLKETSFNQAYGRDLMEAQEWCRKYMKSGNV<br>KDLLQAWDLYYHVFRRISK
	FRB:Cul1(ΔC501)	pcDNA3.1-FRB:gs10:Cul1(ΔC501)
		ASRILWHEMWHEGLEEASRLYFGERNVKGMFEVLEPLHAMMERGPQTLKETSFNQAYGRDLMEAQE<br>WCRKYMKSGNVKDLLQAWDLYYHVFRRISKGGSGGGSGGSSKLQRMFQDIGVSKDLNEQFKKHLTN<br>SEPLDLDFSIQVLSSGSWPFQQSCTFALPSELERSYQRFTAFYASRHSGRKLTWLYQLSKGELVTN<br>CFKNRYTLQASTFQMAILLQYNTEDAYTVQQLTDSTQIKMDILAQVLQILLKSKLLVLEDENANVD<br>EVELKPDTLIKLYLGYKNKKLRVNINVPMKTEQKQEQETTHKNIEEDRKLLIQAAIVRIMKMRKVL<br>KHQQLLGEVLTQLSSRFKPRVPVIKKCIDILIEKEYLERVDGEKDTYSYLA
	Rbx1:FRB	pcDNA3.1-Rbx1: gs10:FRB
		AAAMDVDTPSGTNSGAGKKRFEVKKWNAVALWAWDIVVDNCAICRNHIMDLCIECQANQASATSEE<br>CTVAWGVCNHAFHFHCISRWLKTRQVCPLDNREWEFQKYGHGGSGGGSGGSASRILWHEMWHEGLE<br>EASRLYFGERNVKGMFEVLEPLHAMMERGPQTLKETSFNQAYGRDLMEAQEWCRKYMKSGNVKDLL<br>QAWDLYYHVFRRISK
	FRB:CDC34	pcDNA3.1-FRB:gs10:CDC34
		ASRILWHEMWHEGLEEASRLYFGERNVKGMFEVLEPLHAMMERGPQTLKETSFNQAYGRDLMEAQE<br>WCRKYMKSGNVKDLLQAWDLYYHVFRRISKGGSGGGSGGSMARPLVPSSQKALLLELKGLQEEPVE<br>GFRVTLVDEGDLYNWEVAIFGPPNTYYEGGYFKARLKFPIDYPYSPPAFRFLTKMWHPNIYETGDV<br>CISILHPPVDDPQSGELPSERWNPTQNVRTILLSVISLLNEPNTFSPANVDASVMYRKWKESKGKD<br>REYTDIIRKQVLGTKVDAERDGVKVPTTLAEYCVKTKAPAPDEGSDLFYDDYYEDGEVEEEADSCF<br>GDDEDDSGTEES
4a	CAR:mCit:FKBP	pcDNA3.1-CD8leader:myc:VL:(G4S)3:VH:CD8hinge:CD8TM:4-1BB:CD3zeta:gsg:mCit:gs10:FKBP
		ALPVTALLLPLALLLHAARPEQKLISEEDLDIQMTQTTSSLSASLGDRVTISCRASQDISKYLNWY<br>QQKPDGTVKLLIYHTSRLHSGVPSRFSGSGSGTDYSLTISNLEQEDIATYFCQQGNTLPYTFGGGT<br>KLEITGGGGSGGGGSGGGGSEVKLQESGPGLVAPSQSLSVTCTVSGVSLPDYGVSWIRQPPRKGLE<br>WLGVIWGSETTYYNSALKSRLTIIKDNSKSQVFLKMNSLQTDDTAIYYCAKHYYYGGSYAMDYWGQ<br>GTSVTVSSTTTPAPRPPTPAPTIASQPLSLRPEACRPAAGGAVHTRGLDFACDIYIWAPLAGTCGV<br>LLLSLVITLYCKRGRKKLLYIFKQPFMRPVQTTQEEDGCSCRFPEEEEGGCELRVKFSRSADAPAY<br>QQGQNQLYNELNLGRREEYDVLDKRRGRDPEMGGKPRRKNPQEGLYNELQKDKMAEAYSEIGMKGE<br>RRRGKGHDGLYQGLSTATKDTYDALHMQALPPRGSGVSKGEELFTGVVPILVELDGDVNGHKFSVS<br>GEGEGDATYGKLTLKFICTTGKLPVPWPTLVTTFGYGLMCFARYPDHMKQHDFFKSAMPEGYVQER<br>TIFFKDDGNYKTRAEVKFEGDTLVNCIELKGIDFKEDGNILGHKLEYNYNSHNVYIMADKQKNGIK<br>VNFKIRHNIEDGSVQLADHYQQNTPIGDGPVLLPDNHYLSYQSALSKDPNEKRDHMVLLEFVTAAG<br>ITLGMDELYKGGSGGGSGGSGVQVETISPGDGRTFPKRGQTCVVHYTGMLEDGKKFDSSRDRNKPF<br>KFMLGKQEVIRGWEEGVAQMSVGQRAKLTISPDYAYGATGHPGIIPPHATLVFDVELLKLE
	FRB:SOCSbox	pcDNA3.1-FRB:gs10:SOCSbox
		ASRILWHEMWHEGLEEASRLYFGERNVKGMFEVLEPLHAMMERGPQTLKETSFNQAYGRDLMEAQE<br>WCRKYMKSGNVKDLLQAWDLYYHVFRRISKGGSGGGSGGSTSAPSLQHLCRLTINKCTGAIWGLPL<br>PTRLKDYLEEYKFQV
	CAR:mCit:FRB	pcDNA3.1-CD8leader:myc:VL:(G4S)3:VH:CD8hinge:CD8TM:4-1BB:CD3zeta:gsg:mCit:gs10:FRB

		ALPVTALLLPLALLLHAARPEQKLISEEDLDIQMTQTTSSLSASLGDRVTISCRASQDISKYLNWY<br>QQKPDGTVKLLIYHTSRLHSGVPSRFSGSGSGTDYSLTISNLEQEDIATYFCQQGNTLPYTFGGGT<br>KLEITGGGGSGGGGSGGGGSEVKLQESGPGLVAPSQSLSVTCTVSGVSLPDYGVSWIRQPPRKGLE<br>WLGVIWGSETTYYNSALKSRLTIIKDNSKSQVFLKMNSLQTDDTAIYYCAKHYYYGGSYAMDYWGQ<br>GTSVTVSSTTTPAPRPPTPAPTIASQPLSLRPEACRPAAGGAVHTRGLDFACDIYIWAPLAGTCGV<br>LLLSLVITLYCKRGRKKLLYIFKQPFMRPVQTTQEEDGCSCRFPEEEEGGCELRVKFSRSADAPAY<br>QQGQNQLYNELNLGRREEYDVLDKRRGRDPEMGGKPRRKNPQEGLYNELQKDKMAEAYSEIGMKGE<br>RRRGKGHDGLYQGLSTATKDTYDALHMQALPPRGSGVSKGEELFTGVVPILVELDGDVNGHKFSVS<br>GEGEGDATYGKLTLKFICTTGKLPVPWPTLVTTFGYGLMCFARYPDHMKQHDFFKSAMPEGYVQER<br>TIFFKDDGNYKTRAEVKFEGDTLVNCIELKGIDFKEDGNILGHKLEYNYNSHNVYIMADKQKNGIK<br>VNFKIRHNIEDGSVQLADHYQQNTPIGDGPVLLPDNHYLSYQSALSKDPNEKRDHMVLLEFVTAAG<br>ITLGMDELYKGGSGGGSGGSASRILWHEMWHEGLEEASRLYFGERNVKGMFEVLEPLHAMMERGPQ<br>TLKETSFNQAYGRDLMEAQEWCRKYMKSGNVKDLLQAWDLYYHVFRRISK
	FKBP:CDC34	pcDNA3.1-FKBP:gs10:CDC34
		GVQVETISPGDGRTFPKRGQTCVVHYTGMLEDGKKFDSSRDRNKPFKFMLGKQEVIRGWEEGVAQM<br>SVGQRAKLTISPDYAYGATGHPGIIPPHATLVFDVELLKLEGGSGGGSGGSMARPLVPSSQKALLL<br>ELKGLQEEPVEGFRVTLVDEGDLYNWEVAIFGPPNTYYEGGYFKARLKFPIDYPYSPPAFRFLTKM<br>WHPNIYETGDVCISILHPPVDDPQSGELPSERWNPTQNVRTILLSVISLLNEPNTFSPANVDASVM<br>YRKWKESKGKDREYTDIIRKQVLGTKVDAERDGVKVPTTLAEYCVKTKAPAPDEGSDLFYDDYYED<br>GEVEEEADSCFGDDEDDSGTEES
	BFP	pcDNA3.1-BFP
		SELIKENMHMKLYMEGTVDNHHFKCTSEGEGKPYEGTQTMRIKVVEGGPLPFAFDILATSFLYGSK<br>TFINHTQGIPDFFKQSFPEGFTWERVTTYEDGGVLTATQDTSLQDGCLIYNVKIRGVNFTSNGPVM<br>QKKTLGWEAFTETLYPADGGLEGRNDMALKLVGGSHLIANAKTTYRSKKPAKNLKMPGVYYVDYRL<br>ERIKEANNETYVEQHEVAVARYCDLPSKLGHKLN
4b	FKBP:fLuc:NLS	pcDNA3.1-FKBP:gs10:fLuc:NLS
		GVQVETISPGDGRTFPKRGQTCVVHYTGMLEDGKKFDSSRDRNKPFKFMLGKQEVIRGWEEGVAQM<br>SVGQRAKLTISPDYAYGATGHPGIIPPHATLVFDVELLKLEGGSGGGSGGSEDAKNIKKGPAPFYP<br>LEDGTAGEQLHKAMKRYALVPGTIAFTDAHIEVDITYAEYFEMSVRLAEAMKRYGLNTNHRIVVCS<br>ENSLQFFMPVLGALFIGVAVAPANDIYNERELLNSMGISQPTVVFVSKKGLQKILNVQKKLPIIQK<br>IIIMDSKTDYQGFQSMYTFVTSHLPPGFNEYDFVPESFDRDKTIALIMNSSGSTGLPKGVALPHRT<br>ACVRFSHARDPIFGNQIIPDTAILSVVPFHHGFGMFTTLGYLICGFRVVLMYRFEEELFLRSLQDY<br>KIQSALLVPTLFSFFAKSTLIDKYDLSNLHEIASGGAPLSKEVGEAVAKRFHLPGIRQGYGLTETT<br>SAILITPEGDDKPGAVGKVVPFFEAKVVDLDTGKTLGVNQRGELCVRGPMIMSGYVNNPEATNALI<br>DKDGWLHSGDIAYWDEDEHFFIVDRLKSLIKYKGYQVAPAELESILLQHPNIFDAGVAGLPDDDAG<br>ELPAAVVVLEHGKTMTEKEIVDYVASQVTTAKKLRGGVVFVDEVPKGLTGKLDARKIREILIKAKK<br>GGKIAVNSDPKKKRKV
	F-box:FRB:NLS	pcDNA3.1-F-box:gs10:FRB:NLS
		RENFPGVSWDSLPDELLLGIFSCLCLPELLKVSGVCKRWYRLASDESLWQTLDLTGKNLHPDVTGR<br>LLSGGSGGGSGGSASRILWHEMWHEGLEEASRLYFGERNVKGMFEVLEPLHAMMERGPQTLKETSF<br>NQAYGRDLMEAQEWCRKYMKSGNVKDLLQAWDLYYHVFRRISKDPKKKRKV
	Skp1(ΔC111):FRB:NLS	pcDNA3.1-Skp1(ΔC111):gs10:FRB:NLS
		PSIKLQSSDGEIFEVDVEIAKQSVTIKTMLEDLGMDDEGDDDPVPLPNVNAAILKKVIQWCTHHKD<br>DPPPPEDDENKEKRTDDIPVWDQEFLKVDQGTLFELILAANYLDIGGSGGGSGGSASRILWHEMWH<br>EGLEEASRLYFGERNVKGMFEVLEPLHAMMERGPQTLKETSFNQAYGRDLMEAQEWCRKYMKSGNV<br>KDLLQAWDLYYHVFRRISKDPKKKRKV
	NLS:FRB:Cul1(ΔC501)	pcDNA3.1-NLS:FRB:gs10:Cul1(ΔC501)
		DPKKKRKVASRILWHEMWHEGLEEASRLYFGERNVKGMFEVLEPLHAMMERGPQTLKETSFNQAYG<br>RDLMEAQEWCRKYMKSGNVKDLLQAWDLYYHVFRRISKGGSGGGSGGSSKLQRMFQDIGVSKDLNE<br>QFKKHLTNSEPLDLDFSIQVLSSGSWPFQQSCTFALPSELERSYQRFTAFYASRHSGRKLTWLYQL<br>SKGELVTNCFKNRYTLQASTFQMAILLQYNTEDAYTVQQLTDSTQIKMDILAQVLQILLKSKLLVL<br>EDENANVDEVELKPDTLIKLYLGYKNKKLRVNINVPMKTEQKQEQETTHKNIEEDRKLLIQAAIVR<br>IMKMRKVLKHQQLLGEVLTQLSSRFKPRVPVIKKCIDILIEKEYLERVDGEKDTYSYLA
	Rbx1:FRB:NLS	pcDNA3.1-Rbx1:gs10:FRB:NLS
		AAAMDVDTPSGTNSGAGKKRFEVKKWNAVALWAWDIVVDNCAICRNHIMDLCIECQANQASATSEE<br>CTVAWGVCNHAFHFHCISRWLKTRQVCPLDNREWEFQKYGHGGSGGGSGGSASRILWHEMWHEGLE<br>EASRLYFGERNVKGMFEVLEPLHAMMERGPQTLKETSFNQAYGRDLMEAQEWCRKYMKSGNVKDLL<br>QAWDLYYHVFRRISKDPKKKRKV
	NLS:FRB:CDC34	pcDNA3.1-NLS:FRB:gs10:CDC34
		DPKKKRKVASRILWHEMWHEGLEEASRLYFGERNVKGMFEVLEPLHAMMERGPQTLKETSFNQAYG<br>RDLMEAQEWCRKYMKSGNVKDLLQAWDLYYHVFRRISKGGSGGGSGGSMARPLVPSSQKALLLELK<br>GLQEEPVEGFRVTLVDEGDLYNWEVAIFGPPNTYYEGGYFKARLKFPIDYPYSPPAFRFLTKMWHP<br>NIYETGDVCISILHPPVDDPQSGELPSERWNPTQNVRTILLSVISLLNEPNTFSPANVDASVMYRK<br>WKESKGKDREYTDIIRKQVLGTKVDAERDGVKVPTTLAEYCVKTKAPAPDEGSDLFYDDYYEDGEV<br>EEEADSCFGDDEDDSGTEES
4c	Skp1(ΔC111):FRB:NLS	Same as in Fig. 4b
	Skp1(ΔC111):FRB:NLS	Same as in Fig. 4b
	NLS:FRB:Cul1(ΔC501)	Same as in Fig. 4b
	Rbx1:FRB:NLS	Same as in Fig. 4b

	NLS:FRB:CDC34	Same as in Fig. 4b
	FKBP:dCas9:VPR	pcDNA3.1- FKBP:gs10:dCas9:NLS:VPR
		GVQVETISPGDGRTFPKRGQTCVVHYTGMLEDGKKFDSSRDRNKPFKFMLGKQEVIRGWEEGVAQM<br>SVGQRAKLTISPDYAYGATGHPGIIPPHATLVFDVELLKLEGGSGGGSGGSDKKYSIGLAIGTNSV<br>GWAVITDEYKVPSKKFKVLGNTDRHSIKKNLIGALLFDSGETAEATRLKRTARRRYTRRKNRICYL<br>QEIFSNEMAKVDDSFFHRLEESFLVEEDKKHERHPIFGNIVDEVAYHEKYPTIYHLRKKLVDSTDK<br>ADLRLIYLALAHMIKFRGHFLIEGDLNPDNSDVDKLFIQLVQTYNQLFEENPINASGVDAKAILSA<br>RLSKSRRLENLIAQLPGEKKNGLFGNLIALSLGLTPNFKSNFDLAEDAKLQLSKDTYDDDLDNLLA<br>QIGDQYADLFLAAKNLSDAILLSDILRVNTEITKAPLSASMIKRYDEHHQDLTLLKALVRQQLPEK<br>YKEIFFDQSKNGYAGYIDGGASQEEFYKFIKPILEKMDGTEELLVKLNREDLLRKQRTFDNGSIPH<br>QIHLGELHAILRRQEDFYPFLKDNREKIEKILTFRIPYYVGPLARGNSRFAWMTRKSEETITPWNF<br>EEVVDKGASAQSFIERMTNFDKNLPNEKVLPKHSLLYEYFTVYNELTKVKYVTEGMRKPAFLSGEQ<br>KKAIVDLLFKTNRKVTVKQLKEDYFKKIECFDSVEISGVEDRFNASLGTYHDLLKIIKDKDFLDNE<br>ENEDILEDIVLTLTLFEDREMIEERLKTYAHLFDDKVMKQLKRRRYTGWGRLSRKLINGIRDKQSG<br>KTILDFLKSDGFANRNFMQLIHDDSLTFKEDIQKAQVSGQGDSLHEHIANLAGSPAIKKGILQTVK<br>VVDELVKVMGRHKPENIVIEMARENQTTQKGQKNSRERMKRIEEGIKELGSQILKEHPVENTQLQN<br>EKLYLYYLQNGRDMYVDQELDINRLSDYDVDAIVPQSFLKDDSIDNKVLTRSDKNRGKSDNVPSEE<br>VVKKMKNYWRQLLNAKLITQRKFDNLTKAERGGLSELDKAGFIKRQLVETRQITKHVAQILDSRMN<br>TKYDENDKLIREVKVITLKSKLVSDFRKDFQFYKVREINNYHHAHDAYLNAVVGTALIKKYPKLES<br>EFVYGDYKVYDVRKMIAKSEQEIGKATAKYFFYSNIMNFFKTEITLANGEIRKRPLIETNGETGEI<br>VWDKGRDFATVRKVLSMPQVNIVKKTEVQTGGFSKESILPKRNSDKLIARKKDWDPKKYGGFDSPT<br>VAYSVLVVAKVEKGKSKKLKSVKELLGITIMERSSFEKNPIDFLEAKGYKEVKKDLIIKLPKYSLF<br>ELENGRKRMLASAGELQKGNELALPSKYVNFLYLASHYEKLKGSPEDNEQKQLFVEQHKHYLDEII<br>EQISEFSKRVILADANLDKVLSAYNKHRDKPIREQAENIIHLFTLTNLGAPAAFKYFDTTIDRKRY<br>TSTKEVLDATLIHQSITGLYETRIDLSQLGGDDPKKKRKVEASGSGRADALDDFDLDMLGSDALDD<br>FDLDMLGSDALDDFDLDMLGSDALDDFDLDMLINTSGGSGGGSGGSSQYLPDTDDRHRIEEKRKRT<br>YETFKSIMKKSPFSGPTDPRPPPRRIAVPSRSSASVPKPAPQPYPFTSSLSTINYDEFPTMVFPSG<br>QISQASALAPAPPQVLPQAPAPAPAPAMVSALAQAPAPVPVLAPGPPQAVAPPAPKPTQAGEGTLS<br>EALLQLQFDDEDLGALLGNSTDPAVFTDLASVDNSEFQQLLNQGIPVAPHTTEPMLMEYPEAITRL<br>VTGAQRPPDPAPAPLGAPGLPNGLLSGDEDFSSIADMDFSALLGSGSGSRDSREGMFLPKPEAGSA<br>ISDVFEGREVCQPKRIRPFHPPGSPWANRPLPASLAPTPTGPVHEPVGSLTPAPVPQPLDPAPAVT<br>PEASHLLEDPDEETSQAVKALREMADTVIPQKEEAAICGQMDLSHPPPRGHLDELTTTLESMTEDL<br>NLDSPLTPELNEILDTFLNDECLLHAMHISTGLSIFDTSLF
	sgRNA A	pgRNA-sgRNA(A):scaffold
		agtagcgggagcagcagtaaagttttagagctagaaatagcaagttaaaataaggctagtccgtta<br>tcaacttgaaaaagtggcaccgagtcggtgcttttttt
	1A-pmin-fLuc2CP	pGL4.16-fLuc2CP
		EDAKNIKKGPAPFYPLEDGTAGEQLHKAMKRYALVPGTIAFTDAHIEVDITYAEYFEMSVRLAEAM<br>KRYGLNTNHRIVVCSENSLQFFMPVLGALFIGVAVAPANDIYNERELLNSMGISQPTVVFVSKKGL<br>QKILNVQKKLPIIQKIIIMDSKTDYQGFQSMYTFVTSHLPPGFNEYDFVPESFDRDKTIALIMNSS<br>GSTGLPKGVALPHRTACVRFSHARDPIFGNQIIPDTAILSVVPFHHGFGMFTTLGYLICGFRVVLM<br>YRFEEELFLRSLQDYKIQSALLVPTLFSFFAKSTLIDKYDLSNLHEIASGGAPLSKEVGEAVAKRF<br>HLPGIRQGYGLTETTSAILITPEGDDKPGAVGKVVPFFEAKVVDLDTGKTLGVNQRGELCVRGPMI<br>MSGYVNNPEATNALIDKDGWLHSGDIAYWDEDEHFFIVDRLKSLIKYKGYQVAPAELESILLQHPN<br>IFDAGVAGLPDDDAGELPAAVVVLEHGKTMTEKEIVDYVASQVTTAKKLRGGVVFVDEVPKGLTGK<br>LDARKIREILIKAKKGGKIAVNSACKNWFSSLSHFVIHLNSHGFPPEVEEQAAGTLPMSCAQESGM<br>DRHPAACASARINV
5b	FKBP:fLuc	Same as in Fig. 3c
	CAR:mCit:FKBP	Same as in Fig. 4a
	FKBP:fLuc:NLS	Same as in Fig. 4b
	FRB:SSD	pcDNA3.1-FRB:gs10:SOCSbox:gs10:Skp1(ΔC111)
		ASRILWHEMWHEGLEEASRLYFGERNVKGMFEVLEPLHAMMERGPQTLKETSFNQAYGRDLMEAQE<br>WCRKYMKSGNVKDLLQAWDLYYHVFRRISKGGSGGGSGGSTSAPSLQHLCRLTINKCTGAIWGLPL<br>PTRLKDYLEEYKFQVGGSGGGSGGSPSIKLQSSDGEIFEVDVEIAKQSVTIKTMLEDLGMDDEGDD<br>DPVPLPNVNAAILKKVIQWCTHHKDDPPPPEDDENKEKRTDDIPVWDQEFLKVDQGTLFELILAAN<br>YLDI
	NLS:FRB:SSD	pcDNA3.1-NLS:FRB:gs10:SOCSbox:gs10:Skp1(ΔC111)
		DPKKKRKVASRILWHEMWHEGLEEASRLYFGERNVKGMFEVLEPLHAMMERGPQTLKETSFNQAYG<br>RDLMEAQEWCRKYMKSGNVKDLLQAWDLYYHVFRRISKGGSGGGSGGSTSAPSLQHLCRLTINKCT<br>GAIWGLPLPTRLKDYLEEYKFQVGGSGGGSGGSPSIKLQSSDGEIFEVDVEIAKQSVTIKTMLEDL<br>GMDDEGDDDPVPLPNVNAAILKKVIQWCTHHKDDPPPPEDDENKEKRTDDIPVWDQEFLKVDQGTL<br>FELILAANYLDI
5c	FKBP:fLuc	Same as in Fig. 3c
	FRB:SSD	Same as in Fig. 5b
5d	FKBP:fLuc	Same as in Fig. 3c
	FRB:SSD	Same as in Fig. 5b
S6	fLuc	pcDNA3.1-fLuc:gsg: HA
		EDAKNIKKGPAPFYPLEDGTAGEQLHKAMKRYALVPGTIAFTDAHIEVDITYAEYFEMSVRLAEAM<br>KRYGLNTNHRIVVCSENSLQFFMPVLGALFIGVAVAPANDIYNERELLNSMGISQPTVVFVSKKGL

		QKILNVQKKLPIIQKIIIMDSKTDYQGFQSMYTFVTSHLPPGFNEYDFVPESFDRDKTIALIMNSS<br>GSTGLPKGVALPHRTACVRFSHARDPIFGNQIIPDTAILSVVPFHHGFGMFTTLGYLICGFRVVLM<br>YRFEEELFLRSLQDYKIQSALLVPTLFSFFAKSTLIDKYDLSNLHEIASGGAPLSKEVGEAVAKRF<br>HLPGIRQGYGLTETTSAILITPEGDDKPGAVGKVVPFFEAKVVDLDTGKTLGVNQRGELCVRGPMI<br>MSGYVNNPEATNALIDKDGWLHSGDIAYWDEDEHFFIVDRLKSLIKYKGYQVAPAELESILLQHPN<br>IFDAGVAGLPDDDAGELPAAVVVLEHGKTMTEKEIVDYVASQVTTAKKLRGGVVFVDEVPKGLTGK<br>LDARKIREILIKAKKGGKIAVNSGSGYPYDVPDYA
	F-box:fLuc	pcDNA3.1-f-box:gs10:fluc:gsg: HA
		RENFPGVSWDSLPDELLLGIFSCLCLPELLKVSGVCKRWYRLASDESLWQTLDLTGKNLHPDVTGR<br>LLSGGSGGGSGGSEDAKNIKKGPAPFYPLEDGTAGEQLHKAMKRYALVPGTIAFTDAHIEVDITYA<br>EYFEMSVRLAEAMKRYGLNTNHRIVVCSENSLQFFMPVLGALFIGVAVAPANDIYNERELLNSMGI<br>SQPTVVFVSKKGLQKILNVQKKLPIIQKIIIMDSKTDYQGFQSMYTFVTSHLPPGFNEYDFVPESF<br>DRDKTIALIMNSSGSTGLPKGVALPHRTACVRFSHARDPIFGNQIIPDTAILSVVPFHHGFGMFTT<br>LGYLICGFRVVLMYRFEEELFLRSLQDYKIQSALLVPTLFSFFAKSTLIDKYDLSNLHEIASGGAP<br>LSKEVGEAVAKRFHLPGIRQGYGLTETTSAILITPEGDDKPGAVGKVVPFFEAKVVDLDTGKTLGV<br>NQRGELCVRGPMIMSGYVNNPEATNALIDKDGWLHSGDIAYWDEDEHFFIVDRLKSLIKYKGYQVA<br>PAELESILLQHPNIFDAGVAGLPDDDAGELPAAVVVLEHGKTMTEKEIVDYVASQVTTAKKLRGGV<br>VFVDEVPKGLTGKLDARKIREILIKAKKGGKIAVNSGSGYPYDVPDYA
	fLuc:Cul1(ΔC501)	pcDNA3.1-HA:gsg:fluc:gs10:Cul1(dC501)
		YPYDVPDYAGSGEDAKNIKKGPAPFYPLEDGTAGEQLHKAMKRYALVPGTIAFTDAHIEVDITYAE<br>YFEMSVRLAEAMKRYGLNTNHRIVVCSENSLQFFMPVLGALFIGVAVAPANDIYNERELLNSMGIS<br>QPTVVFVSKKGLQKILNVQKKLPIIQKIIIMDSKTDYQGFQSMYTFVTSHLPPGFNEYDFVPESFD<br>RDKTIALIMNSSGSTGLPKGVALPHRTACVRFSHARDPIFGNQIIPDTAILSVVPFHHGFGMFTTL<br>GYLICGFRVVLMYRFEEELFLRSLQDYKIQSALLVPTLFSFFAKSTLIDKYDLSNLHEIASGGAPL<br>SKEVGEAVAKRFHLPGIRQGYGLTETTSAILITPEGDDKPGAVGKVVPFFEAKVVDLDTGKTLGVN<br>QRGELCVRGPMIMSGYVNNPEATNALIDKDGWLHSGDIAYWDEDEHFFIVDRLKSLIKYKGYQVAP<br>AELESILLQHPNIFDAGVAGLPDDDAGELPAAVVVLEHGKTMTEKEIVDYVASQVTTAKKLRGGVV<br>FVDEVPKGLTGKLDARKIREILIKAKKGGKIAVNSGGSGGGSGGSSKLQRMFQDIGVSKDLNEQFK<br>KHLTNSEPLDLDFSIQVLSSGSWPFQQSCTFALPSELERSYQRFTAFYASRHSGRKLTWLYQLSKG<br>ELVTNCFKNRYTLQASTFQMAILLQYNTEDAYTVQQLTDSTQIKMDILAQVLQILLKSKLLVLEDE<br>NANVDEVELKPDTLIKLYLGYKNKKLRVNINVPMKTEQKQEQETTHKNIEEDRKLLIQAAIVRIMK<br>MRKVLKHQQLLGEVLTQLSSRFKPRVPVIKKCIDILIEKEYLERVDGEKDTYSYLA
	Rbx1:fLuc	pcDNA3.1-Rbx1:gs10:fluc:gsg: HA
		AAAMDVDTPSGTNSGAGKKRFEVKKWNAVALWAWDIVVDNCAICRNHIMDLCIECQANQASATSEE<br>CTVAWGVCNHAFHFHCISRWLKTRQVCPLDNREWEFQKYGHGGSGGGSGGSEDAKNIKKGPAPFYP<br>LEDGTAGEQLHKAMKRYALVPGTIAFTDAHIEVDITYAEYFEMSVRLAEAMKRYGLNTNHRIVVCS<br>ENSLQFFMPVLGALFIGVAVAPANDIYNERELLNSMGISQPTVVFVSKKGLQKILNVQKKLPIIQK<br>IIIMDSKTDYQGFQSMYTFVTSHLPPGFNEYDFVPESFDRDKTIALIMNSSGSTGLPKGVALPHRT<br>ACVRFSHARDPIFGNQIIPDTAILSVVPFHHGFGMFTTLGYLICGFRVVLMYRFEEELFLRSLQDY<br>KIQSALLVPTLFSFFAKSTLIDKYDLSNLHEIASGGAPLSKEVGEAVAKRFHLPGIRQGYGLTETT<br>SAILITPEGDDKPGAVGKVVPFFEAKVVDLDTGKTLGVNQRGELCVRGPMIMSGYVNNPEATNALI<br>DKDGWLHSGDIAYWDEDEHFFIVDRLKSLIKYKGYQVAPAELESILLQHPNIFDAGVAGLPDDDAG<br>ELPAAVVVLEHGKTMTEKEIVDYVASQVTTAKKLRGGVVFVDEVPKGLTGKLDARKIREILIKAKK<br>GGKIAVNSGSGYPYDVPDYA
	fLuc:CDC34	pcDNA3.1- HA:gsg:fluc:gsg:CDC34
		YPYDVPDYAGSGEDAKNIKKGPAPFYPLEDGTAGEQLHKAMKRYALVPGTIAFTDAHIEVDITYAE<br>YFEMSVRLAEAMKRYGLNTNHRIVVCSENSLQFFMPVLGALFIGVAVAPANDIYNERELLNSMGIS<br>QPTVVFVSKKGLQKILNVQKKLPIIQKIIIMDSKTDYQGFQSMYTFVTSHLPPGFNEYDFVPESFD<br>RDKTIALIMNSSGSTGLPKGVALPHRTACVRFSHARDPIFGNQIIPDTAILSVVPFHHGFGMFTTL<br>GYLICGFRVVLMYRFEEELFLRSLQDYKIQSALLVPTLFSFFAKSTLIDKYDLSNLHEIASGGAPL<br>SKEVGEAVAKRFHLPGIRQGYGLTETTSAILITPEGDDKPGAVGKVVPFFEAKVVDLDTGKTLGVN<br>QRGELCVRGPMIMSGYVNNPEATNALIDKDGWLHSGDIAYWDEDEHFFIVDRLKSLIKYKGYQVAP<br>AELESILLQHPNIFDAGVAGLPDDDAGELPAAVVVLEHGKTMTEKEIVDYVASQVTTAKKLRGGVV<br>FVDEVPKGLTGKLDARKIREILIKAKKGGKIAVNSGSGMARPLVPSSQKALLLELKGLQEEPVEGF<br>RVTLVDEGDLYNWEVAIFGPPNTYYEGGYFKARLKFPIDYPYSPPAFRFLTKMWHPNIYETGDVCI<br>SILHPPVDDPQSGELPSERWNPTQNVRTILLSVISLLNEPNTFSPANVDASVMYRKWKESKGKDRE<br>YTDIIRKQVLGTKVDAERDGVKVPTTLAEYCVKTKAPAPDEGSDLFYDDYYEDGEVEEEADSCFGD<br>DEDDSGTEES
	Skp1(ΔC111):fLuc	pcDNA3.1-Skp1(dc111): gs10:fluc:gsg: HA
		PSIKLQSSDGEIFEVDVEIAKQSVTIKTMLEDLGMDDEGDDDPVPLPNVNAAILKKVIQWCTHHKD<br>DPPPPEDDENKEKRTDDIPVWDQEFLKVDQGTLFELILAANYLDIGGSGGGSGGSEDAKNIKKGPA<br>PFYPLEDGTAGEQLHKAMKRYALVPGTIAFTDAHIEVDITYAEYFEMSVRLAEAMKRYGLNTNHRI<br>VVCSENSLQFFMPVLGALFIGVAVAPANDIYNERELLNSMGISQPTVVFVSKKGLQKILNVQKKLP<br>IIQKIIIMDSKTDYQGFQSMYTFVTSHLPPGFNEYDFVPESFDRDKTIALIMNSSGSTGLPKGVAL<br>PHRTACVRFSHARDPIFGNQIIPDTAILSVVPFHHGFGMFTTLGYLICGFRVVLMYRFEEELFLRS<br>LQDYKIQSALLVPTLFSFFAKSTLIDKYDLSNLHEIASGGAPLSKEVGEAVAKRFHLPGIRQGYGL<br>TETTSAILITPEGDDKPGAVGKVVPFFEAKVVDLDTGKTLGVNQRGELCVRGPMIMSGYVNNPEAT<br>NALIDKDGWLHSGDIAYWDEDEHFFIVDRLKSLIKYKGYQVAPAELESILLQHPNIFDAGVAGLPD<br>DDAGELPAAVVVLEHGKTMTEKEIVDYVASQVTTAKKLRGGVVFVDEVPKGLTGKLDARKIREILI<br>KAKKGGKIAVNSGSGYPYDVPDYA
S7	P3:fLuc:HA	pcDNA3.1-P3:gs10:fLuc:gsg:HA
		SPEDEIQQLEEEIAQLEQKNAALKEKNQALKYGGGSGGGSGGSEDAKNIKKGPAPFYPLEDGTAGE<br>QLHKAMKRYALVPGTIAFTDAHIEVDITYAEYFEMSVRLAEAMKRYGLNTNHRIVVCSENSLQFFM

		PVLGALFIGVAVAPANDIYNERELLNSMGISQPTVVFVSKKGLQKILNVQKKLPIIQKIIIMDSKT<br>DYQGFQSMYTFVTSHLPPGFNEYDFVPESFDRDKTIALIMNSSGSTGLPKGVALPHRTACVRFSHA<br>RDPIFGNQIIPDTAILSVVPFHHGFGMFTTLGYLICGFRVVLMYRFEEELFLRSLQDYKIQSALLV<br>PTLFSFFAKSTLIDKYDLSNLHEIASGGAPLSKEVGEAVAKRFHLPGIRQGYGLTETTSAILITPE<br>GDDKPGAVGKVVPFFEAKVVDLDTGKTLGVNQRGELCVRGPMIMSGYVNNPEATNALIDKDGWLHS<br>GDIAYWDEDEHFFIVDRLKSLIKYKGYQVAPAELESILLQHPNIFDAGVAGLPDDDAGELPAAVVV<br>LEHGKTMTEKEIVDYVASQVTTAKKLRGGVVFVDEVPKGLTGKLDARKIREILIKAKKGGKIAVNS<br>GSGYPYDVPDYA
	F-box:P4	Same as in Fig. 2b
	Skp1(ΔC111):P4	Same as in Fig. 2b
	P4:Cul1(ΔC501)	Same as in Fig. 2b
	Rbx1:P4	Same as in Fig. 2b
	P4:CDC34	Same as in Fig. 2b
S8	P3:fLuc:HA	Same as in Fig. S7
	F-box:P4	Same as in Fig. 2a
	Skp1(ΔC111):P4	Same as in Fig. 2a
	P4:Cul1(ΔC501)	Same as in Fig. 2a
	Rbx1:P4	Same as in Fig. 2a
	P4:CDC34	Same as in Fig. 2a
S10	FKBP:fLuc	Same as in Fig. 3c
	fLuc:FKBP	Same as in Fig. 3c
	FKBP:fLuc:NLS	Same as in Fig. 4b
	FKBP:dCas9:VPR	Same as in Fig. 4c
	sgRNA A	Same as in Fig. 4c
	1A-pmin-fLuc2CP	Same as in Fig. 4c

References

1 Zheng N, Schulman BA, Song L, Miller JJ, Jeffrey PD, Wang P, Chu C, Koepp DM, Elledge SJ, Pagano M, Conaway RC, Conaway JW, Harper JW & Pavletich NP (2002) Structure of the Cul1–Rbx1–Skp1–F box Skp2 SCF ubiquitin ligase complex. *Nature* **416**, 703–709.

2 Schulman BA, Carrano AC, Jeffrey PD, Bowen Z, Kinnucan ERE, Finnin MS, Elledge SJ, Harper JW, Pagano M & Pavletich NP (2000) Insights into SCF ubiquitin ligases from the structure of the Skp1-Skp2 complex. *Nature* **408**, 381–386.

3 Bullock AN, Debreczeni JE, Edwards AM, Sundstrom M & Knapp S (2006) Crystal structure of the SOCS2-elongin C-elongin B complex defines a prototypical SOCS box ubiquitin ligase. *Proc Natl Acad Sci* **103**, 7637–7642.
